# Reproducibility of F18‐FDG PET radiomic features for different cervical tumor segmentation methods, gray‐level discretization, and reconstruction algorithms

**DOI:** 10.1002/acm2.12170

**Published:** 2017-09-11

**Authors:** Baderaldeen A. Altazi, Geoffrey G. Zhang, Daniel C. Fernandez, Michael E. Montejo, Dylan Hunt, Joan Werner, Matthew C. Biagioli, Eduardo G. Moros

**Affiliations:** ^1^ Department of Radiation Oncology H.L. Moffitt Cancer Center and Research Institute Tampa FL USA; ^2^ Department of Physics University of South Florida Tampa FL USA; ^3^ Department of Radiation Oncology King Fahad Specialist Hospital Dammam Saudi Arabia; ^4^ Department of Radiation Oncology Florida Hospital Orlando FL USA

**Keywords:** cervical cancer, FDG, gray‐level discretization, MTV, PET, radiomics

## Abstract

Site‐specific investigations of the role of radiomics in cancer diagnosis and therapy are emerging. We evaluated the reproducibility of radiomic features extracted from ^18^Flourine–fluorodeoxyglucose (^18^F‐FDG) PET images for three parameters: manual versus computer‐aided segmentation methods, gray‐level discretization, and PET image reconstruction algorithms. Our cohort consisted of pretreatment PET/CT scans from 88 cervical cancer patients. Two board‐certified radiation oncologists manually segmented the metabolic tumor volume (MTV_1_ and MTV_2_) for each patient. For comparison, we used a graphical‐based method to generate semiautomated segmented volumes (GBSV). To address any perturbations in radiomic feature values, we down‐sampled the tumor volumes into three gray‐levels: 32, 64, and 128 from the original gray‐level of 256. Finally, we analyzed the effect on radiomic features on PET images of eight patients due to four PET 3D‐reconstruction algorithms: maximum likelihood‐ordered subset expectation maximization (OSEM) iterative reconstruction (IR) method, fourier rebinning‐ML‐OSEM (FOREIR), FORE‐filtered back projection (FOREFBP), and 3D‐Reprojection (3DRP) analytical method. We extracted 79 features from all segmentation method, gray‐levels of down‐sampled volumes, and PET reconstruction algorithms. The features were extracted using gray‐level co‐occurrence matrices (GLCM), gray‐level size zone matrices (GLSZM), gray‐level run‐length matrices (GLRLM), neighborhood gray‐tone difference matrices (NGTDM), shape‐based features (SF), and intensity histogram features (IHF). We computed the Dice coefficient between each MTV and GBSV to measure segmentation accuracy. Coefficient values close to one indicate high agreement, and values close to zero indicate low agreement. We evaluated the effect on radiomic features by calculating the mean percentage differences ($\bar{d}$) between feature values measured from each pair of parameter elements (i.e. segmentation methods: MTV_1_‐MTV_2_, MTV_1_‐GBSV, MTV_2_‐GBSV; gray‐levels: 64‐32, 64‐128, and 64‐256; reconstruction algorithms: OSEM‐FORE‐OSEM, OSEM‐FOREFBP, and OSEM‐3DRP). We used $|\bar{d}|$ as a measure of radiomic feature reproducibility level, where any feature scored $|\bar{d}|$ ±SD ≤ |25|% ± 35% was considered reproducible. We used Bland–Altman analysis to evaluate the mean, standard deviation (SD), and upper/lower reproducibility limits (U/LRL) for radiomic features in response to variation in each testing parameter. Furthermore, we proposed U/LRL as a method to classify the level of reproducibility: *High*— ±1% ≤ U/LRL ≤ ±30%; *Intermediate*— ±30% < U/LRL ≤ ±45%; *Low*— ±45 < U/LRL ≤ ±50%. We considered any feature below the *low* level as nonreproducible (NR). Finally, we calculated the interclass correlation coefficient (ICC) to evaluate the reliability of radiomic feature measurements for each parameter. The segmented volumes of 65 patients (81.3%) scored Dice coefficient >0.75 for all three volumes. The result outcomes revealed a tendency of higher radiomic feature reproducibility among segmentation pair MTV_1_‐GBSV than MTV_2_‐GBSV, gray‐level pairs of 64‐32 and 64‐128 than 64‐256, and reconstruction algorithm pairs of OSEM‐FOREIR and OSEM‐FOREFBP than OSEM‐3DRP. Although the choice of cervical tumor segmentation method, gray‐level value, and reconstruction algorithm may affect radiomic features, some features were characterized by high reproducibility through all testing parameters. The number of radiomic features that showed insensitivity to variations in segmentation methods, gray‐level discretization, and reconstruction algorithms was 10 (13%), 4 (5%), and 1 (1%), respectively. These results suggest that a careful analysis of the effects of these parameters is essential prior to any radiomics clinical application.

## 1 INTRODUCTION

Radiological imaging in oncology is becoming essential in daily clinical practice. Therefore, the focus has shifted toward comprehensive quantification of radiological image data. This process would allow for the extraction of more useful underlying information based on quantitatively derived features: Radiomics. Several institutes have reported quantitative analysis studies, with a focus on radiomic features, for different imaging modalities such as computed tomography (CT),^1^, ^2^, ^3^ and magnetic resonance imaging (MRI).^4^, ^5^, ^6^ The investigation of positron emission tomography (PET) radiomics was first reported in 2009.^7^, ^8^, ^9^ In recent years, fluorine‐18‐labeled fluoro‐2‐deoxy‐d‐glucose positron emission tomography–computed tomography, ^18^F–FDG (PET/CT) has become a major functional imaging technique in oncology due to its ability to evaluate tumor stage and metabolic characteristics with high specificity and sensitivity.^10^, ^11^

Since the start of ^18^F–FDG PET clinical application, there has been a rapid growth in the number of studies that employed standardized uptake value (SUV) as a primary imaging biomarker for uptake heterogeneity quantification. Such studies employed maximum, mean and peak SUVs (SUV_Max_, SUV_Mean_, and SUV_Peak_, respectively) as biomarkers for prediction,^12^ diagnosis, and monitoring of treatment response.^13^ While SUV_Max_ and SUV_Mean_ have been widely studied, SUV_Peak_ has recently been reported by Sher et al^14^ The latter is defined as the maximum of all the mean values computed from placing a spherical kernel of approximately 1.2 cm in diameter to yield a ~1 cm^3^ sphere centered at each voxel within the tumor volume.^15^ In addition to SUV measurements, metabolic tumor volume (MTV) is another biomarker that has been reportedly shown to have prognostic significance for clinical outcomes such as the development of distant metastasis and loco‐regional recurrence.^16^ Some studies have demonstrated the ability of MTV to quantify heterogeneity of PET uptake in the detection of pelvic lymph nodes in cervical cancer^17^, ^18^ as well as in the association with treatment response within the same site.^19^ Other studies focused on investigating the performance of SUV to predict for survival endpoints or treatment outcomes of cervical cancer, and head‐and‐neck tumors.^20^ However, relying solely on semiquantitative measurements, SUV or MTV, as biomarkers have been shown to run into several pitfalls. For example, in addition to radiotracer dose sensitivity, SUV measurements are highly influenced by the distribution of radiotracer uptake, delayed time of injection, and imaging acquisition and reconstruction parameters.^21^ These factors can potentially reflect in substantial treatment assessment uncertainty.

As an alternative, several studies^22^ proposed quantitative imaging features, such as radiomic features, as a surrogate to overcome such pitfalls. Textural features, a type of radiomic features, are extracted from statistical matrices based on local intensity spatial distribution relationships. They are thought to be independent of tumor size, position, and time of imaging.^23^ These characteristics made textural features superior to SUV measurements regarding tumor heterogeneity characterization. Also, shape features (SF), which describe geometrical characteristics of tumors, have shown to provide a morphological characterization of PET uptake heterogeneity within a specified volume of interest.^24^, ^25^ Recent studies have emphasized on the higher discriminatory power of several radiomic features in comparison to SUV measurements regarding classification of tumor versus benign regions in lung, and head‐and‐neck patients,^26^ as well as for the prediction of cervical cancer treatment outcomes.^27^ Radiomic features were also reported as a significant tool to stage cervical cancer based on tumor heterogeneity information.^28^ Along the same line, Cheng et al^29^ reported that uniformity, a GLCM feature, might serve as an independent prognostic predictor as well as risk stratification descriptor for patients with oropharyngeal squamous cell carcinoma. Another study investigated the physiologic reproducibility of textural features by characterizing the tumor F18‐FDG uptake heterogeneity in the PET scans of 41 esophageal cancer patients.^30^

All the findings mentioned above indicates that quantitative assessment of tumor uptake heterogeneity based on PET ^18^F–FDG images is a promising method to investigate intra‐ and inter‐tumor characteristics. With such encouraging results, the focus is shifting toward examining the reproducibility of radiomic features due to various factors that might potentially affect their performance. The most challenging of these factors is the definition of tumor volume.

An extensive review study by Foster et al^31^ identified five sophisticated procedures of PET tumor segmentation, namely manual segmentation, thresholding‐based methods, learning methods and stochastic modeling‐based techniques, region‐based (graphical‐based) segmentation methods, and boundary‐based methods. The study concluded that there is no notion of one acceptable PET image segmentation method over the other. Also, it was suggested that further research is needed to come to a conclusion of an optimal method for PET segmentation. For more studies with efforts to enhance methods of tumor segmentation on PET scans, the reader is encouraged to review these articles.^32^, ^33^, ^34^, ^35^ In this study, we explored the differences between using graphical‐ and boundary‐based methods in comparison to the manual method for segmenting the cervical tumor volumes on PET scans.

Since the introduction of tomographic reconstruction application to medical imaging in late 1960s, research work has progressed to enhance image formation. In recent years, varying reconstruction methods have evolved into sophisticated algorithms with various image qualities due to modern computing. A study by Galavis et al^36^ showed that different acquisition modes and image reconstruction settings might cause variation in radiomic features. Similarly, the gray‐level discretization of PET/CT images has shown to have a great impact on some radiomic features.^37^

All the mentioned studies investigated the reproducibly of radiomic features in different body sites. To our knowledge, the reproducibility of radiomic features in cervical cancer tumors has not been widely reported. Thus, the purpose of this work was to investigate the sensitivity of radiomic features with regard to three critical parameters: segmentation methods, gray‐levels discretization, and PET reconstruction algorithms. The rationale was to develop a group of radiomic features that might serve as robust biomarkers for cervical cancer outcome assessment.

## 2 METHODS

### 2.A Patient demographics and scanner specifications

Our dataset consisted of pretreatment PET/CT scans from a cohort of 88 patients diagnosed with cervical cancer (age range: 31–76 yr). We used 80 patients for segmentation methods, and gray‐level testing and 8 for reconstruction algorithm testing. All patients were treated with external beam radiation therapy to a dose ranging between 45 and 50.4 Gy (median dose of 45 Gy), concurrent cisplatin chemotherapy and MRI‐planned brachytherapy to a dose of 20–30 Gy (median total dose of 28 Gy). The patients’ disease was staged according to the International Federation of Gynecology and Obstetrics (FIGO) classification.^38^, ^39^ The number of patients with FIGO stages IB, IIA, IIB, IIIA, and IIIB were 24, 37, 12, and 15, respectively.

This research study acquired the approval of our institutional review board (IRB) at the University of South Florida. All of the patients’ pretreatment PET/CT scans were performed in the Radiology Department of Moffitt Cancer Center on the same Discovery STE^®^ hybrid PET/CT scanner (*General Electric Medical Systems, Milwaukee, WI, USA*)^40^ and under the same institutional F18‐FDG administration protocol. PET images had a slice thickness of 3.30 mm and spatial resolution of 5.49 × 5.49 mm/pixel and were acquired after 60 min of injection with 6 MBq/kg of 18F‐FDG. The PET images were reconstructed using 3D maximum likelihood‐ordered subsets expectation maximization (ML–OSEM) with two iterations and 28 subsets. All of PET images were corrected for attenuation and then converted to SUV units (g/ml).

### 2.B Method of tumor segmentation

In a measurement error study, we often consider the observers as a random sample from a larger population of potential observers who may be used in future studies or clinical practice.^41^ In the present study, we treated each segmentation method as a different observer of the tumor volume. In this case, we were not interested in drawing conclusions about the performance of a particular segmentation method, the observer, but only in the information provided by the effect of their variation on radiomic features.

For the purpose of segmentation methods and gray‐level effect analysis, two board‐certified radiation oncologists manually delineated the metabolic tumor volume (MTV_1_, MTV_2_) in the uterus and cervix regions based on the F–18 FDG uptake in pretreatment PET scans. The oncologists utilized CT scans and patient‐specific histopathological reports for guidance to differentiate between cervix, bladder, and other surrounding organs. Both MTVs were generated using Mirada Medical DBx^®^, Oxford, UK. Due to the lack of a ground truth for tumors, we chose MTV_1_ to be the reference, gold standard, tumor volume due to the physician's experience. Subsequently, we generated semiautomated graphical‐based volumes (GBSV) based on the method reported by Beichel et al^42^ For further information about this method, the reader is encouraged to review the cited article. This approach is implemented as an extension for 3D Slicer software (https://www.slicer.org/), an open source software package to visualize and analyze medical images. We studied the effects of RA variation on radiomic features extracted from the GBSVs.

### 2.C Method of gray intensity level discretization

This preprocessing step is essential as the value of the extracted radiomic features varies widely from each other. Also, it helps to reduce image noise by normalizing intensities across all patients’ images or tumor volumes. Therefore, it allows for a direct comparison of all calculated radiomic features among patients. To investigate the effect of gray‐level, image intensity values, discretization on radiomic features, we down‐sampled the tumor volumes for each patient into three gray‐levels, 32, 64, and 128, in addition to the original 256. Using such fixed numbers of discrete resampled values, number of bins, divides the image SUV range into equally spaced intervals. Therefore, the bin sizes, intensity resolutions, of the discretized volumes depended on the SUV range (i.e., four bin sizes for each gray‐level) as indicated by Eq. (1):

(1) $$\text{Bin Size}=\frac{SUV_{\max}-SUV_{\min}}{N_{g}}$$

where Ng: the number of gray‐level bins.

### 2.D PET reconstruction algorithms

**Figure 1:**
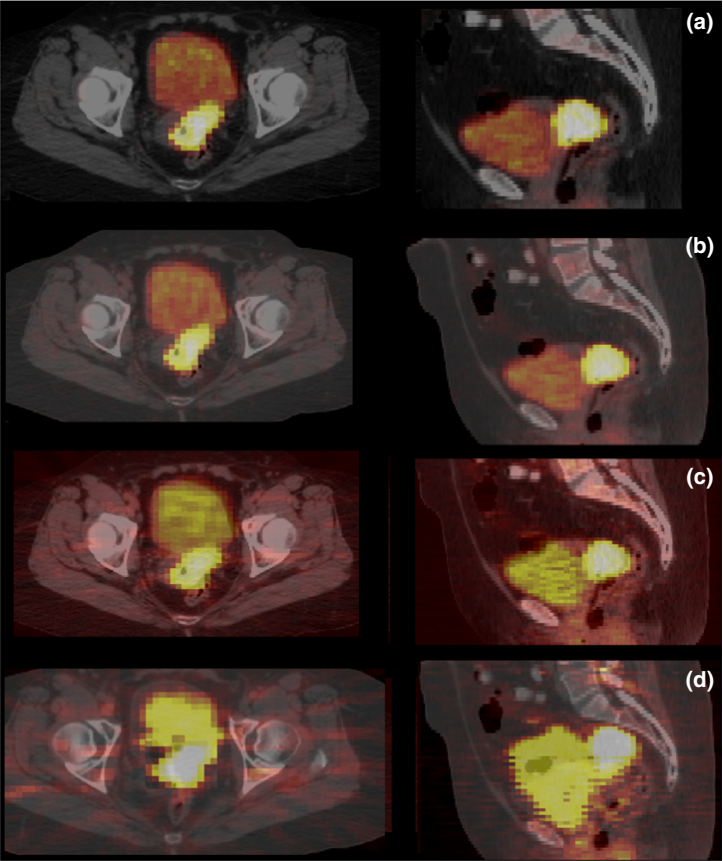
PET image variations due to different reconstruction algorithms (RA): (a) *Maximum Likelihood‐Ordered Subset Expectation Maximization Iterative (IR) Method (*ML‐OSEM*)*, (b) *Fourier Rebinning‐ML‐OSEM (*FOREIR*)*, (c) *FORE‐Filtered Back Projection (*FORE FBP*)*, and (d) *Three‐Dimensional Reprojection (*3DRP*)*.

One of the goals of this study was to focus on the effect of common PET reconstruction algorithms on radiomic features, but not to discuss the difference between them. For references on medical image reconstruction, the interested reader is encouraged to read.^43^, ^44^ In addition to ML‐OSEM, the conventional iterative reconstruction (IR) algorithm in GE Discovery STE scanners, we explored the impact of three additional reconstruction settings (Fig. 1) on radiomic features: Fourier rebinned FOREIR, FORE‐filtered backprojection reconstruction (FOREFBP), and three‐dimensional reprojection algorithm (3DRP).

### 2.E Radiomics analysis

In a recent study, Kumar et al defined radiomics as^45^ “the extraction and analysis of large amounts of advanced high throughput of imaging features with high throughput from medical images obtained with computed tomography, positron emission tomography or magnetic resonance imaging. Importantly, these data are designed to be extracted from standard‐of‐care images, leading to a considerable potential subject pool”. Radiomic features can be divided into different categories according to their method of feature extraction. The most common ones are textural and shape features. The intensity arrangements found in a region of interest (ROI) within an image can have various patterns, which can hold valuable information about the ROI (e.g., tumor volume). These patterns are often called a *texture*. A *textural feature* of a radiological image describes the spatial relationships among the gray intensity levels of voxels; *textural analysis* is, therefore, the mathematical extraction of textural features and their subsequent correlation of biological or clinical variables. On the other hand, shape‐based features are calculated to describe the morphological characteristics of ROIs. Recently, all the mentioned quantitative imaging features are referred to as radiomic features. We developed in‐house software to process and quantify PET scans, and also to calculate the five commonly implemented methods of feature extraction. In total, we extracted 79 radiomic features according to the following methods.

#### 2.E.1 Feature extraction using gray‐level co‐occurrence matrix

The gray‐level co‐occurrence matrix (GLCM) (also known as spatial gray‐level sensitivity matrix) is a second‐order statistical method that characterizes the local information of gray‐levels between pairs of voxels; hence, the extracted features are considered *local* features. In our implementation, the relationships between consecutive neighboring voxels in 13 directions in a three‐dimensional space were quantified using a one‐voxel displacement vector between a voxel and its neighbor (i.e., voxel offest is one in all directions). Twenty‐six features were calculated using this method.^23^ GLCM features have become one of the most well‐known and widely used texture features. Examples of this approach are *Second‐order Entropy, Difference Entropy, Inverse Difference (ID), Inverse Difference Moment (IDM), and Information Measure of Correlation (IMC)*.

#### 2.E.2 Feature extraction using gray‐level run‐length matrix

Gray‐level run‐length matrix (GLRLM) were used to extract 11 *regional* features, which captures the coarseness characteristics of image textures in specific directions within the predefined segmented volume.^46^ A *run* is defined as the length of consecutive voxels that share the same gray‐level intensity along a specific linear direction. This method was mainly applied to generate features based on fine textures that tend to contain more short runs with similar gray‐level intensities; and coarse textures, which tend to have more long runs with significantly different gray intensities level. Examples of this method are: *Short Run Emphasis SRE* (measures the distribution of short runs in the image), *Long Run Emphasis LRE (*measures the distribution of long runs in the image), and *Run Percentage RPC (*measures the homogeneity and the distribution of runs of an image in a specific direction).

#### 2.E.3 Feature extraction using gray‐level size zone matrix

Gray‐level size zone matrix (GLSZM) also extract 11 *regional* features. However, the method of extraction takes place by quantifying the clusters of homogenous intensity regions within the tumor.^47^ Examples of this approach are *High‐Intensity Emphasis (HIE), Low‐Intensity Emphasis (LIE), Size Zone Variability (SZV), Small Area Emphasis (SAE), and Large Area Emphasis (LAE)*.

#### 2.E.4 Feature extraction using neighborhood gray‐tone difference matrix method

We calculated this set of features according to the method initially proposed by Amadasun and King.^48^ The five neighborhood gray‐tone difference matrix method (NGTDM) features are thought to mimic human visual impressions. Note that the original NGTDM feature equations were defined only for square ROIs. However, the calculations were modified slightly to apply them to irregularly shaped, and multiple slice ROIs in 3D space. We used a neighborhood of 7 × 7 pixels for all PET images in this study. The five higher order features were *coarseness, contrast, complexity, busyness,* and *texture strength*.

#### 2.E.5 Feature extraction using shape aspects

We extracted six shape‐based features (SF) to describe morphological and geometrical aspects of tumor volumes. Examples of this method are *convexity* (a measure of tumors solidity), *eccentricity* (a measure of noncircularity of tumors), and the *ratio of tumor surface area to tumor volume* (Surf/Vol).^10^, ^49^

#### 2.E.6 Feature extraction using intensity–volume histogram

Tumor volume was plotted as a function of the image intensity to generate *global* features. We calculated twenty common first‐order metrics such as the *mean, standard deviation, maximum and minimum intensities, skewness,* and *kurtosis*. We also studied other intensity–volume histogram (IVH)‐based features reported by El Naqa et al^27^ Examples of such features are *V90* (volume percentage having at least intensity of 90%) and *I90* (minimum intensity of 90% of the highest intensity volume).

### 2.F Statistical analysis

As previously mentioned, MTV_1_ was chosen as the reference volume. To assess segmentation accuracy, we computed the Dice coefficient between the semiautomatic and manual segmentations. For the segmented volumes MTV_1_, MTV_2,_ and GBSV, the Dice coefficient is given by the following equation:

(2) $$\text{DC}=\left(2|{\text{MTV}}_{1\;\mathrm{OR}\;2}\cap \text{GBSV}|\right)\;/\;\left(|{\text{MTV}}_{1\;\mathrm{OR}\;2}\left|+|\text{GBSV}\right|\right)$$

To assess the level of agreement between both experts; we reported the Dice coefficient based on their observation for the same tumor.

**Figure 2:**
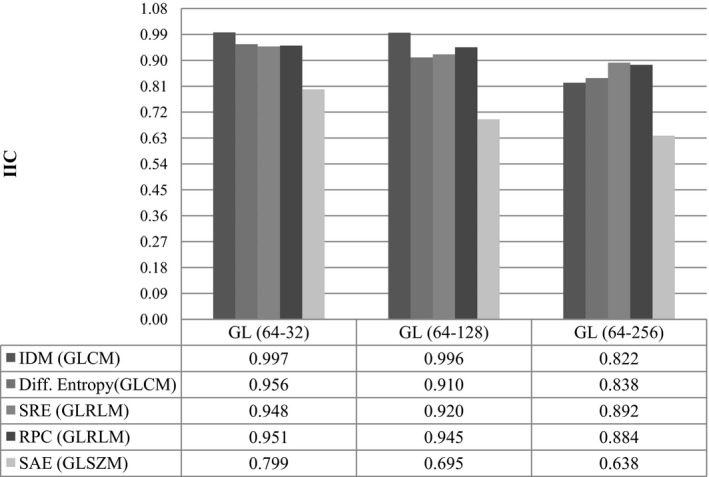
Inter‐item correlation coefficient (IIC) among Reference gray‐level 64 (GL‐64) relative to GL‐32,128,256. The plots show IIC of several radiomic features extracted from the metabolic tumor volume (MTV) after down‐sampling. It is noticed that IIC is minimum between GL 64 and GL 256. GLCM, Gray Level Co‐occurrence Matrix; GLRLM, Gray Level Run Length Matrix; GLSZM, Gray Level Size Zone Matrix; IDM, Inverse Difference Moment; Diff. Entropy, Difference Entropy; SRE, Short Run Emphasis; RPC, Run Percentage; SAE, Size Area Emphasis.

We generated matrices of inter‐item correlation coefficients (IIC)^50^, ^51^, ^52^ to determine the reference gray‐level. As gray‐level 64 demonstrated the highest IIC in comparison with all gray‐levels (Fig. 2). We investigated the reproducibility of each feature through the first parameter by pairing the reference tumor volume with the other two volumes (MTV_1_–MTV_2_ and MTV_1_‐GBSV). For the second parameter, we paired each of the three distinct gray‐levels with the reference (64‐32, 64‐128, and 64‐256) and for the third, we paired ML‐OSEM with FORE‐OSEM, FOREFBP, and 3DRP. We studied and reported each test separately. We expressed the difference between radiomic feature values measured from each element of the testing parameters by the mean percentage difference $|\bar{d}|$ (Eq. (3)):

(3) $$|\bar{d}|=\left|\frac{f_{m}-f_{n}}{(f_{m}+f_{n})/2}\times 100\%\right|$$

where f_m_ and f_n_ represent features extracted from first and second segmentation methods, gray‐level or reconstruction algorithms. Bland–Altman analysis is a graphical method to quantify the agreement between two quantitative measurements by studying the mean difference within which 95% of the difference between the second measure in comparison to the first measure fall.^53^ We used the Bland–Altman analysis to evaluate the mean, standard deviation (SD), and upper/lower reproducibility limits (URL/LRL), Eqs. (4) and (5), for radiomic features in response to variation in each testing parameter.^54^, ^55^, ^56^

(4) URL=Mean+(1.96×SD)

(5) LRL=Mean−(1.96×SD)

The bias between measurements is often estimated by the mean difference ($\bar{d}$) and its associated standard deviation (SD). In this study, we used $|\bar{d}|$ as an indicator for radiomic feature reproducibility level, where any feature scored $|\bar{d}|$ ± SD ≤ |25|% ± 35% was considered reproducible. Furthermore, we proposed the use of U/LRL as criteria to classify the level of reproducibility: *High*— ±1% ≤ U/LRL ≤ ±30%; *Intermediate*— ±30% < U/LRL ≤ ±45%; *Low*— ±45 < U/LRL ≤ ±50%. We considered any feature below the *low* level as nonreproducible (NR). We based this approach on methods reported in several clinical studies.^41^, ^53^, ^54^, ^55^ Also, Galavis et al^36^ used a similar scale to categorize the features based on their variation, and Tixier et al^37^ indicated that such limits were referenced to previously defined reproducibility limits for standard uptake values.

Finally, we calculated the interclass correlation coefficient (ICC) to evaluate the reliability of radiomic feature measurements from each parameter. A perfect agreement is indicated by an ICC value of 1.0. The 95% confidence intervals were also calculated. The precision of ICC (Eq. (6)) served as a basis for evaluating the reproducibility of measurements in each case.^50^

(6) $$\begin{array}{cc} \text{Precision} & =half\;width\;of\;CI\times 100\\ & =\left[(95\%CIUB-95\%CILB)/2\right]\times 100 \end{array}$$

where CI represents confidence interval; UB and LB represent upper and lower bounds, respectively. We considered a *P*‐value of less than 0.05 as statistically significant for all the tests in this study. Finally, we explored the method used in Shafiq et al^57^ to correct for the dependency of radiomic features on voxel size (volume) and gray‐level discretization. All statistical analyses were performed using SPSS (Version 22; IBM Corporation; Armonk, New York, USA) and MedCalc Statistical Software version 17.6 (MedCalc Software, Ostend, Belgium; http://www.medcalc.org; 2017).

## 3 RESULTS

The GBSVs of 65 patients (81.25% of the cohort) scored Dice coefficients >0.75 when associated with both manual segmentations, yet the association with MTV_1_ was slightly higher (4% higher on average). Table S1 shows the detailed segmentation accuracies categorized based on increasing values of MTV_1_. As noticed from the table, Dice coefficients were low for both small (volume ≤~15 cm^3^) and large tumors (volume ≥160 cm^3^). For a fair comparison, we only included highly accurate tumor volumes (DC >0.75, *n* = 65). Finally, we reported the results of gray‐level discretization based on resampling the GBSVs. The following subsections, we will report the reproducibility of radiomic features through each testing parameter separately.

### 3.A Reproducibility of radiomic features through segmentation methods

**Figure 3:**
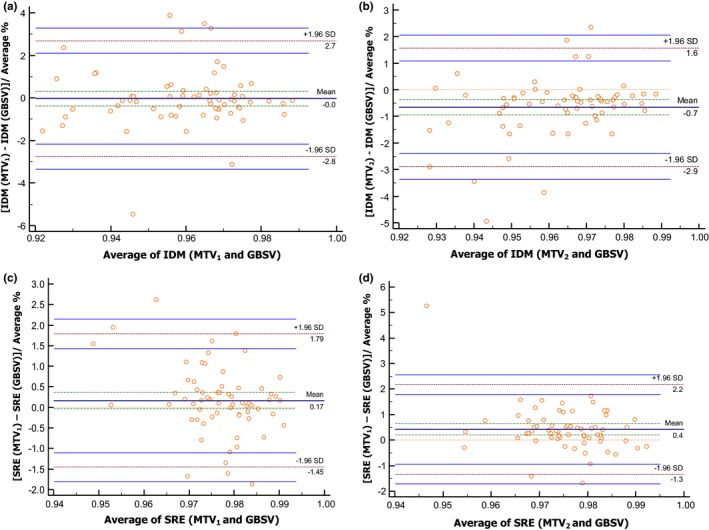
Bland–Altman plots for two of the most reproducible radiomic features through manual vs. the semiautomatic segmentation graphical‐based methods (GBSV). Bland–Altman plots for: (a‐b) Inverse Difference Moment IDM (MTV_1_ – GBSV) and (MTV_2_ – GBSV). (c–d) Short Run Emphasis SRE (MTV_1_ – GBSV) and (MTV_2_ – GBSV).

Among the 26 *local heterogeneity features* extracted using GLCM (Table S2a), eight features (31%) showed high reproducibility, two (7%) showed intermediate reproducibility, and two (7%) showed low reproducibility. The rest of GLCM features (55%) were not reproducible. We also found that IDM holds the highest reproducibility among all methods (Table 1, Figs. 3a and 3b). IDM scored an ICC of 0.90 (Table 2) with a precision of 5%. The ICCs, associated 95% CIs, and precision are summarized in Table 2.

Out of the 11 regional features extracted using GLRLM (Table S2b), only three features (27%) were reproducible. Short run emphasis (SRE) (Figs. 3c and 3d) had the highest reproducibility and ICC (Table 2) of 0.89 with a precision 6.5%.

Among the seven *shape‐based Radiomic features* (Table S2e), four (57%) had high reproducibility, while one (14%) showed low reproducibility. *Tumor volume sphericity* showed high reproducibility through segmentation methods. *Spherical disproportionality* was also reproducible with test outputs close to the one for *tumor volume sphericity*.

All the 11 regional features extracted using *GLSZM* showed high sensitivity to variation in segmentation methods. However, *High‐Intensity Emphasis* HIE and *Zone Percentage* ZP (Table S2c) were the only features to show intermediate reproducibility after correction for gray‐level dependence. Only one *IVH* features (Tables S2f and S2g), *Intensity entropy*, showed high reproducibility. Finally, all the NGTDM features showed high sensitivity to segmentation methods. The highly reproducible features are summarized in Table 1.

**Table 1:** Bland–Altman table for the highest reproducible radiomic features as a function of segmentation methods (SM)

	MTV_1_ – GBSV			MTV_2_ – GBSV		
Feature	|$\bar{d}$|% ± SD%	LRL	URL	|$\bar{d}$|% ± SD%	LRL	URL
IDM	0.03 ± 2.11	−2.76	2.69	1.20 ± 2.18	−2.89	1.57
ID	0.10 ± 2.49	−5.17	4.97	2.20 ± 5.41	−5.29	2.89
Summation Entropy^a^	0.54 ± 25.15	−23.16	21.56	1.08 ± 14.52	−11.01	9.92
Entropy^a^ (second order)	0.03 ± 18.43	−24.14	22.93	0.79 ± 15.01	−10.87	10.41
Mean^b^ (second order)	2.08 ± 3.57	−13.35	9.18	3.85 ± 2.54	−17.45	9.75
IMC2	3.37 ± 6.61	18.31	16.91	3.02 ± 10.21	−20.06	14.03
SRE	0.17 ± 2.89	−1.45	1.79	0.42 ± 2.24	−1.33	2.18
RPC	1.16 ± 10.43	−10.01	7.69	1.84 ± 8.09	−9.14	10.96
Sph. D	6.31 ± 3.89	−13.53	26.15	5.01 ± 3.50	−15.80	25.81

LRL and URL, lower and upper reproducibility limits, respectively; ID, IDM: Inverse Difference, and Inverse Difference Moment; IMC.1,2, Information Correlation Method 1, and 2 (GLCM); SRE, Short Run Emphasis (GLRLM); RPC, Run Percentage (GLRLM); Sph. D., Spherical Disproportionality (SBF).

a Corrected for volume and gray‐level dependence.

b Corrected for volume and gray‐level dependence.

### 3.B Reproducibility of radiomic features for different numbers of gray‐levels

The goal of this part of the study was to measure reproducibility limits and absolute agreement between radiomic features extracted from multiple gray‐levels of the down‐sampled GBSVs. Following the same approach in the previous subsection, fewer features passed this testing parameter in contrast to the first one.

**Figure 4:**
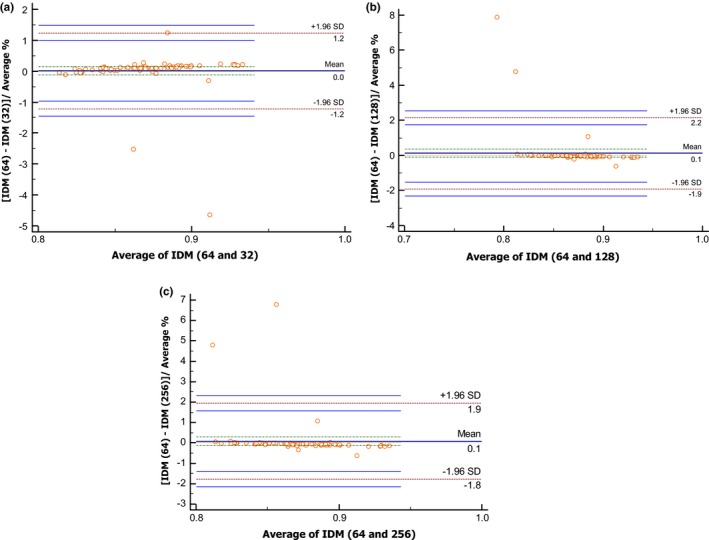
Demonstration of the reproducibility of radiomics through different gray levels (GL). Bland–Altman plot of Inverse Difference Moment (IDM), which is extracted from the resampled semiautomatic graphical‐based segmentation volumes (GBSV) to: (a) GL 64‐32, (b) GL 64‐128, and (c) GL 64‐256.

Among GLCM features, two (18%) were highly reproducible through all gray intensity levels. *IDM* (Table 3, Figs. 4a–4c) was the highest reproducible GLCM feature with a mean difference ± SD of 0.1 ± 2.7 and L/URL range below 6% (ICC 0.98; precision <6%). *Entropy*,* difference entropy* , and *summation entropy* (Table S3a) showed high reproducible levels among gray‐level pairs of 64‐32 and 64‐128 but intermediate reproducibility for gray‐level pair 64‐256. *Second‐order mean* showed intermediate reproducibility after correction for volume and gray‐level dependence. *Dissimilarity* scored was the only feature to show low reproducibility. The rest of GLCM features (69%) were not reproducible. The ICCs, associated 95% CIs, and precision are summarized in Table 4.

**Table 2:** Reliability of radiomic features through segmentation methods SM using ICC

Type	Feature	ICC	95% UCI	95% LCI	Precision
Local	Entropy^a^	0.97	0.95	0.98	±1.5%
	Summation entropy^a^	0.81	0.70	0.88	±9%
	IMC 2	0.73	0.67	0.82	±7.5%
	Mean^b^	0.91	0.86	0.94	±4%
	IDM	0.90	0.84	0.94	±5%
	ID	0.85	0.72	0.92	±10%
	Difference entropy^a^	0.84	0.75	0.89	±7%
Regional	RPC	0.92	0.84	0.92	±4%
	SRE	0.93	0.80	0.93	±6.5%
	LRE	0.89	0.78	0.89	±5.5%
	HIE	0.73	0.58	0.81	±11.5%
Shape & intensity	Sph. D.	0.92	0.86	0.95	±4.5%
	Sphericity	0.91	0.85	0.94	±4.5%
	Intensity entropy	0.85	0.79	0.92	±6.5%
	Convexity	0.70	0.59	0.82	±11.5%

ID, IDM, Inverse Difference, and Inverse Difference Moment; IMC1, 2: Information Correlation Method 1 and 2; RPC, Run Percentage; SRE, Short Run Emphasis; HIE, High‐Intensity Emphasis; Sph. D., Spherical Disproportionality.

a Corrected for GL dependence.

b Corrected for GL and volume dependence.

Among GLRLM features, two (18%) were highly reproducible. In concordance with the result from the previous test, SRE and RPC (Table S3b) were the highest reproducible GLRLM features. The rest of GLRLM features (54.5%) were not reproducible.

None of the GLSZM or NGTDM features showed reproducibility limits range compared to other calculation methods. Hence, all of them were considered sensitive to gray‐level discretization.

**Figure 5:**
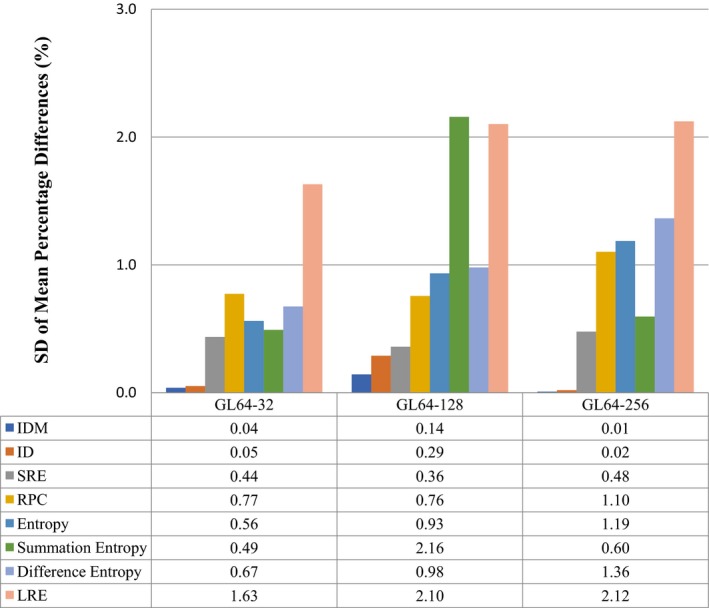
A plot of the standard deviation (SD) of the mean percentage difference ($\bar{d}$) for the top seven reproducible radiomic features as a function of discretization. IDM, Inverse Difference Moment; ID, Inverse Difference; SRE, Short Run Emphasis; RPC, Run Percentage; LRE, Long Run Emphasis.

We did not test shape‐based features or intensity histogram features for gray‐level dependence as the down‐sampled tumor volume was fixed and, therefore, the shape and geometrical aspects were not affected. In general, radiomic features have small mean percentage difference (Fig. 5), SD, and L/URL among gray‐level pairs 64‐32 and 64‐128 in contrast to 64‐256.

### 3.C Reproducibility of radiomic features through different PET reconstruction algorithms

**Figure 6:**
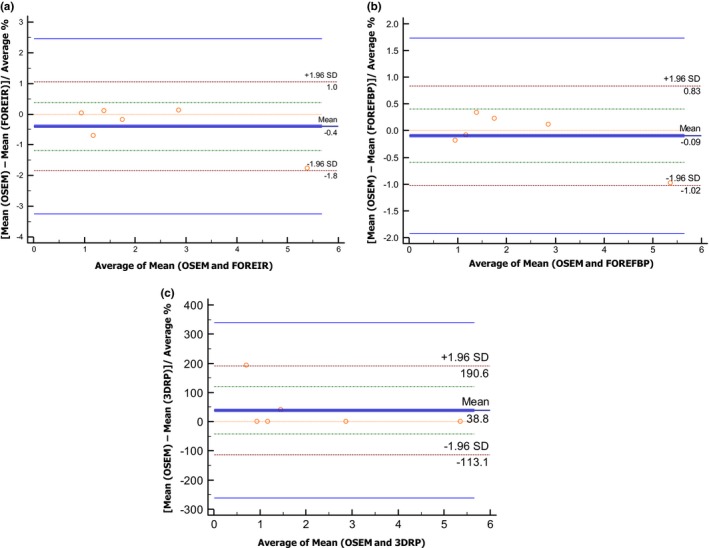
Bland–Altman plots for second‐order mean (GLCM) as a function of reconstruction algorithms (RA). The plots demonstrate the trend of high reproducibility between RA pairs OSEM‐FOREIR and OSEM‐FOREFBP, but low reproducibility for the RA pair OSEM‐3DRP. This trend was noticed for the majority of radiomic features. ML‐OSEM, Maximum Likelihood‐Ordered Subset Expectation Maximization Iterative (IR) Method; FOREIR, Fourier Rebinning‐ML‐OSEM; FORE FBP, FORE‐Filtered Back Projection; 3DRP, Three‐Dimensional Reprojection.

**Figure 7:**
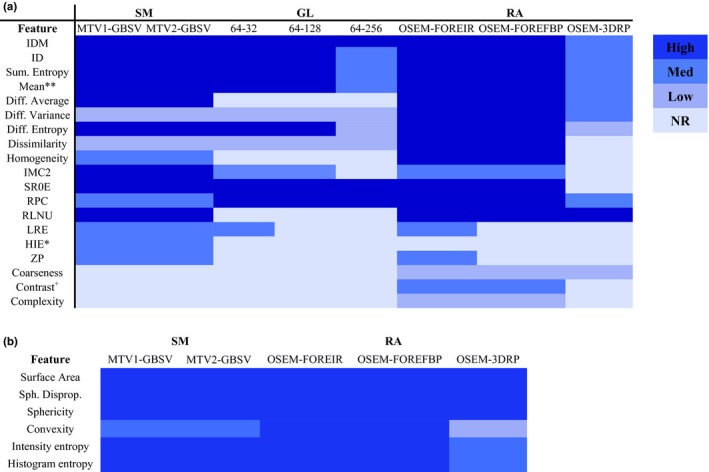
(a) Local (second order) and regional (higher order) radiomic features that showed reproducibility through all testing parameter (SM: Segmentation Method, GL: Gray Level and RA: Reconstruction Algorithm). IDM*,* Inverse Difference Moment*;* ID*, Inverse Difference; IMC2,* Information Measure of Correlation II*;* SRE*,* S*hort Run Emphasis;* RPC*, Run Percentage; RLNU, Run Length Non‐Uniformity;* LRE*,* Long Run Emphasis*; HIE, High Intensity Emphasis; Zp, Zone Percentage*. (b) Shape‐based radiomic features (SF) that showed reproducibility through SM and RA. GL was not included because it does not affect shape‐based features. *Sph. Disprop, Spherical disproportionality*. *Corrected for grey‐level discretization with (a) for GL dependence. **Corrected for gray‐level discretization and voxel size with (b) corrected for GL and volume dependence.

In comparison to the segmentation methods and gray‐levels, radiomic features showed highest variations as a function of reconstruction settings. Following the same evaluation approach used for the previous parameters, some of the features that presented small variations for this parameter (Tables S4a–S4d) are the *entropy*,* second‐order mean,* exclude coarseness, complexity, and contrast. Figure 6 shows *second‐order mean* as an example of such performance. More than twenty features showed a large range of variations, some of these include *HIE, GLNU, texture strength* and *busyness,* which have been commonly used in previous clinical studies. Most of the radiomic feature within the scope of this study showed high sensitivity to 3DRP reconstruction algorithm, the highest reproducible features are listed in Table 5. The reliability of radiomic features through reconstruction algorithms using ICC are found in Table 6. The nine reproducible radiomic features through all parameters are summarized in the colored‐coded map in Fig. 7a. In addition, the six‐reproducible shape feature through segmentation methods and reconstruction algorithms are summarized in Fig. 7b.

**Table 3:** Bland–Altman table for the highest radiomic features as a function of gray intensity levels (GL) for the graph‐based segmented volume (GBSV)

Feature	GL pairs	$|\bar{d}|$% ± SD%	LRL	URL	Level of reproducibility
IDM	64‐32	0.01 ± 0.04	−1.22	1.23	High
	64‐128	0.12 ± 0.29	−1.92	2.15	
	64‐256	0.15 ± 0.02	−1.78	1.94	
ID	64‐32	0.04 ± 0.230	−1.22	1.23	High
	64‐128	0.2 ± 0.144	−1.92	2.15	
	64‐256	0.15 ± 0.07	−1.78	1.94	
SRE	64‐32	2.38 ± 4.03	−0.01	4.78	High
	64‐128	1.19 ± 3.32	−2.67	0.29	
	64‐256	1.87 ± 4.40	−3.53	−0.22	
Mean^b^	64‐32	0.93 ± 15.35	−34.58	36.45	Intermediate
	64‐128	3.58 ± 6.50	−39.32	41.48	
	64‐256	6.08 ± 10.73	−42.00	45.06	
RPC	64‐32	3.62 ± 7.14	0.09	7.14	High
	64‐128	1.49 ± 7.01	−5.02	2.02	
	64‐256	2.30 ± 10.20	−6.92	2.31	
Entropy^a^	64‐32	0.63 ± 0.08	−10.57	11.82	High
	64‐128	9.82 ± 0.03	−9.19	28.84	
	64‐256	5.78 ± 1.81	−30.62	42.17	Intermediate
Summation entropy^a^	64‐32	18.43 ± 5.50	7.84	29.44	High
	64‐128	13.50 ± 8.57	−30.44	3.43	
	64‐256	21.97 ± 10.74	−43.16	−0.80	Intermediate
Difference entropy^a^	64‐32	0.69 ± 6.03	−8.87	7.48	High
	64‐128	9.82 ± 8.76	9.19	28.84	
	64‐256	5.80 ± 12.20	−30.97	42.58	Intermediate

LRL and URL, lower and upper reproducibility limits, respectively; ID, IDM, Inverse Difference, and Inverse Difference Moment; RPC, Run Percentage; SRE, Short Run Emphasis.

a Corrected for GL dependence.

b Corrected for volume and gray‐level dependence.

**Table 4:** Reliability of radiomic features through gray intensity levels GL using ICC

Type	Feature	ICC	95% UCI	95% LCI	Precision
Local	IDM	0.96	0.93	0.98	±2.5%
	ID	0.92	0.90	0.94	±2%
	IMC 2	0.72	0.65	0.90	±12.5%
	Mean^b^	0.85	0.82	0.94	±6%
	Entropy^a^	0.72	0.75	0.83	±4%
	Summation entropy^a^	0.70	0.67	0.79	±6%
	Difference entropy^a^	0.81	0.67	0.86	±9.5%
Regional	RPC	0.89	0.73	0.91	±9%
	LRE	0.72	0.64	0.87	±11.5%
	SRE	0.80	0.70	0.83	±6.5%

ID, IDM, Inverse Difference, and Inverse Difference Moment; IMC1, 2, Information Correlation Method 1 and 2; RPC, Run Percentage; SRE, Short Run Emphasis; LRE, Long Run Emphasis.

a Corrected for GL dependence.

b Corrected for GL and volume dependence.

**Table 5:** Bland–Altman table for the highest radiomic features as a function of PET image reconstruction algorithms (RA)

Feature	RA pairs	Mean ± SD	LRL	URL	Level of reproducibility
IDM	OSEM‐FOREIR	−0.04 ± 15.67	−0.34	0.37	High
	OSEM‐FOREFBP	−0.31 ± 10.34	−0.26	0.26	
	OSEM‐3DRP	−1.02 ± 10.17	−0.14	0.014	Intermediate
ID	OSEM‐FOREIR	0.15 ± 0.99	−0.44	0.76	High
	OSEM‐FOREFBP	−0.31 ± 0.97	−0.54	0.52	
	OSEM‐3DRP	4.33 ± 1.42	−0.49	0.21	Intermediate
SRE	OSEM‐FOREIR	−0.09 ± 0.48	−4.25	11.46	High
	OSEM‐FOREFBP	0.83 ± 3.37	−8.76	4.10	
	OSEM‐3DRP	−23.04 ± 8.18	−12.42	4.82	NR
RPC	OSEM‐FOREIR	−0.30 ± 8.24	−7.88	19.94	High
	OSEM‐FOREFBP	−0.22 ± 20.25	−17.77	9.70	
	OSEM‐3DRP	−25.61 ± 31.60	−26.45	13.56	Intermediate
Mean^b^	OSEM‐FOREIR	−0.29 + 8.87	−1.30	0.73	High
	OSEM‐FOREFBP	−4.92 + 8.45	−15.36	5.52	
	OSEM‐3DRP	1.96 + 8.08	−28.55	32.47	Intermediate
Entropy^a^	OSEM‐FOREIR	−1.44 ± 16.66	8.90	28.65	High
	OSEM‐FOREFBP	−0.39 ± 17.22	−30.28	3.19	
	OSEM‐3DRP	−29.47 ± 17.99	−43.05	−1.17	NR
Summation entropy	OSEM‐FOREIR	−9.06 ± 14.29	7.84	29.44	High
	OSEM‐FOREFBP	−10.61 ± 14.55	−30.44	3.43	
	OSEM‐3DRP	−19.35 ± 10.74	−43.16	−0.80	Intermediate
Difference entropy^a^	OSEM‐FOREIR	5.31 ± 10.01	6.00	30.06	High
	OSEM‐FOREFBP	8.93 ± 10.62	−30.72	4.42	
	OSEM‐3DRP	10.72 ± 19.07	−45.84	2.75	Intermediate
IMC2	OSEM‐FOREIR	−1.41 ± 2.01	−5.44	32.48	High
	OSEM‐FOREFBP	−1.29 ± 2.47	−31.98	3.31	
	OSEM‐3DRP	4.96 ± 3.82	−51.87	3.15	Intermediate
LRE	OSEM‐FOREIR	1.21 ± 0.48	−54.19	21.87	High
	OSEM‐FOREFBP	0.83 ± 3.37	−19.52	39.58	
	OSEM‐3DRP	−23.04 ± 8.18	−22.01	54.70	NR

LRL and URL, lower and upper reproducibility limits, respectively; ID, IDM, Inverse Difference, and Inverse Difference Moment; IMC, Information Correlation; RPC, Run Percentage; SRE, Short Run Emphasis; LRE, Long Run Emphasis.

a Entropy and difference entropy are corrected for GL dependence.

b Corrected for volume and gray‐level dependence.

**Table 6:** Reliability of radiomic features through reconstruction algorithms RA using ICC (3DRP is excluded)

Type	Feature	ICC	95% UCI	95% LCI	Precision
Local	IDM	0.94	0.80	0.98	±9%
	ID	0.97	0.90	0.99	±4.5%
	IMC 2	0.77	0.45	0.89	±22%
	Mean^b^	0.83	0.79	0.94	±6.5%
	Entropy^a^	0.84	0.68	0.96	±14%
	Summation entropy^a^	0.84	0.70	0.96	±13%
	Difference entropy^a^	0.78	0.76	0.95	±9.5%
Regional	RPC	0.90	0.74	0.97	±11.5%
	LRE	0.73	0.65	0.80	±7.5%
	SRE	0.82	0.73	0.94	±10.5%

ID, IDM, Inverse Difference, and Inverse Difference Moment; IMC1,2.: Information Correlation Method 1 and 2; RPC, Run Percentage; SRE, Short Run Emphasis.

a Corrected for GL dependence.

b Corrected for GL and volume dependence.

## 4 DISCUSSION

The promise of radiomics, as with other ‐omics, is the provision of robust markers for personalized medicine applications. One of its potential applications might be in predicting and tracking clinical outcomes for various therapy modalities. Mu et al^28^ observed a high association between textural features on baseline ^18^F–FDG PET and tumor staging in cervical cancer. The study focused on primary tumor volumes because of the limited resolution of PET images, which did not reproduce significant heterogeneity in small lymph nodes. On a similar note, El Naqa et al^27^ reported several logistic regression models of radiomic features, with good prediction power, for cervical cancer treatment outcomes. However, it was suggested that further testing and validation using large datasets is required. Although the use of radiomic features as markers for prediction of treatment outcomes, tumor staging or monitoring response is a rising application of F18‐FDG PET; investigating the reproducibility, reliability, and robustness of such markers through physiological or physical parameters have shown to be a step of great importance. Several image parameters pose unique challenges in the process of quantifying and extracting useful information from the tumor's FDG uptake.^58^ In the present study, we explored the effect of three of these challenging parameters, segmentation methods, gray intensity levels, and reconstruction algorithms, on radiomic features extracted from pretreatment ^18^F‐FDG PET scans of cervical cancer patients.

According to our results, we found that segmentation of cervical tumors revealed challenges due to difficulty in isolating the tumor from adjacent organs, such as bladder and rectum, with similar signal intensities on PET and CT scans. This finding is concordant with another study by Wei et al^59^ To examine the impact of cervical tumor volume variations on radiomic features, we employed two manual volumes segmented by two expert radiation oncologists and one semiautomatic segmented volume. The just‐enough‐interaction (JEI) graphical‐based semiautomatic segmentation approach offered minimal operator interaction and a high degree of automation. To measure the accuracy of tumor segmentation, we overlapped the voxel intensity maps of each tumor pair and calculated the Dice coefficient as a measure of segmentation similarity. As MTV_1_ was closer to GBSV in most cases, the majority of radiomic features showed slightly higher (4%) reproducibility between MTV_1_‐GBSV than MTV_2_‐GBSV. The detailed comparison is found in Table S1.

**Figure 8:**
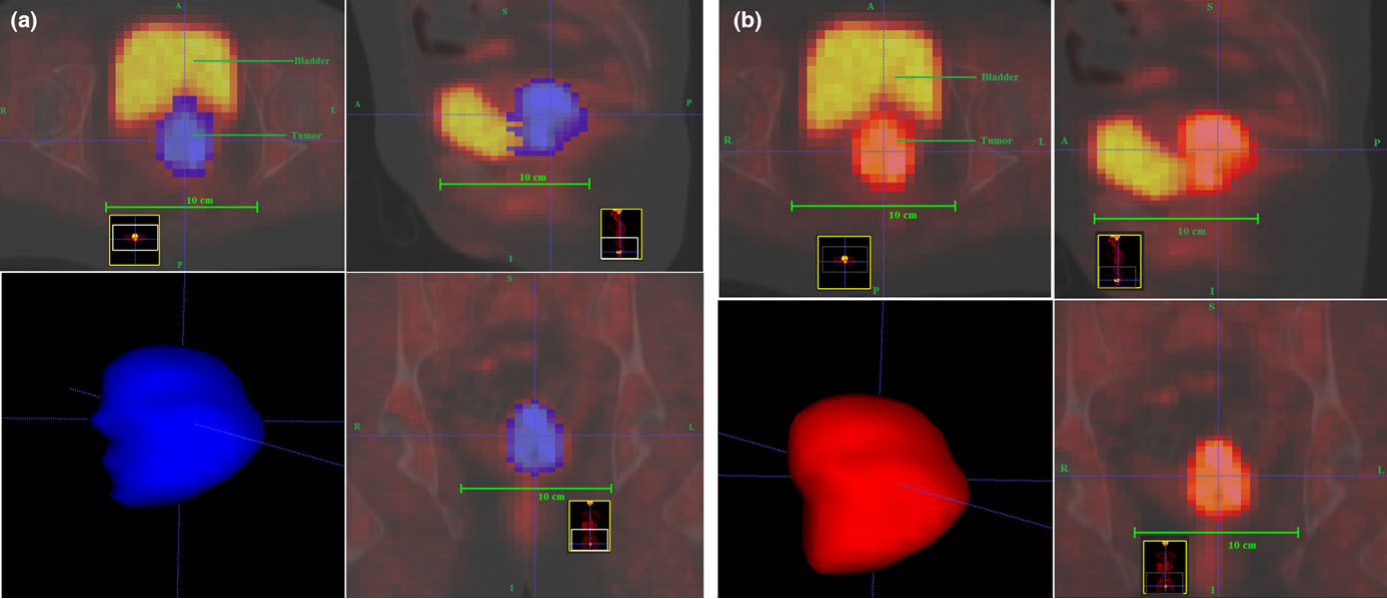
Comparison between: (a) geodesic active contour method with a classification speed function (blue volume), and (b) semiautomatic graphical‐based method (GBSV) for the same tumor volume (red volume). In contrast to GBSV, the geodesic active contour method often captures intensity signals from surrounding organs such as the bladder.

In addition to graphical‐based methods, we also explored the performance of a boundary‐based method called the geodesic active contours, which was first introduced by Caselles et al^60^ We implemented this approach using an open source software called ITK‐SNAP.^61^ A major challenge of such method is to set several equation parameters, especially the speed function. We tested all four methods available for forming speed functions, which are thresholding, classification, clustering, and edge detection methods. Threshold‐based method, as the name implies, utilize the intensities probabilities based on the intensity histogram of the image. For more information calculating the probabilities for this method, the reader is encouraged to read Zhu et al^62^ and Yushkevich et al^61^ A major disadvantage of thresholding method is that the intensity histogram does not provide spatial information about the ROIs. Also, there is no consensus on the selection of an optimum threshold level because of the large variability of pathologies, low resolution, inherent noise, and high uncertainties in fuzzy object boundaries.^31^ Moreover, defining tumor volumes based on SUV thresholds has been widely challenged.^63^, ^64^ As a requirement for the supervised classification‐based method, we trained three labels based on image intensity (1: tumor volume, 2: bladder, and 3: other surrounding tissue) on a training set and applied the resulted classifier on a test set. A disadvantage of using supervised methods is that they do not incorporate spatial information into the decision of label generation. Also, this method required much manual interaction to obtain a training data. Therefore, it is both labor‐intensive and time‐consuming. Nonetheless, the segmentation using this speed function for patients with volumes between 49 to 100 cm^3^ was acceptable (DC>0.75). Otherwise, the volumes highly varied when compared with manual volumes. In contrast to classification method, the clustering method is an unsupervised method that does not require training labels (classes). Edge detection speed functions are given by the image grayscale gradient, where the volume of interest is separated from the surround object in the image by edges, i.e., strong intensity discontinuities. The main limitations of the edge‐based contours are its leakage past weak edges in proximity with surrounding organs and its long processing time. The tumor volumes generated using clustering and edge methods captured intensities from both bladder and rectum, which resulted in highly variant tumor volumes. Within the framework of this study, the only method with just enough interaction that showed high accuracy in comparison to the manual segmented volume was the graphical‐based method. In addition, it was the only method to show full separation between the tumor and the adjacent organ with minimum, if any, operator involvement (Fig. 8). However, this method might need improvement for small tumor volume (≤16 cm^3^) with low uptake, and large volume (≥160 cm^3^) with very high uptake with proximity to surrounding organs.

*Inverse difference moment* IDM (Figs. 3a and 3b, 4a–4c) and *Inverse Difference* ID were the most reproducible through all testing parameters. ID and IDM measure the level of local homogeneity within the tumor volume. Their methods of calculation are based on assuming larger values for smaller gray‐tone differences in pair elements within the gray‐level co‐occurrence matrices (GLCM).^23^, ^66^ Also, they are formulated to have a maximum value when all elements in the image are of equal values. Therefore, these features are characterized by high sensitivity to the presence of adjacent diagonal elements in the GLCM.^65^, ^66^ These characteristics might lead to their remarkable insensitivity toward variation of the studied parameters.

We noticed that the tumor heterogeneity patterns could be profoundly affected by choice of gray‐level. We found higher reproducibility among small gray intensity level pairs (64‐32 and 64‐128) in contrast to lower reproducibility for gray‐level pair 64‐256 (Table S2) in local features (GLCM) and regional features (GLRLM and GLSZM). We noticed that excluding GL‐256 would increase the precision of ICC by ~35%. Also, when resampling the voxel values within the segmented tumor volume to a high gray‐level value, the elements on the GLCM, GLRLM, GLSZM, and NTGDM would read small voxel intensity values relative to the values measured from the reference gray‐level. This trend is consistent with the one reported by Sassi et al^67^ Consequently, this trend yields large mean percentage differences between feature values measured based on different gray‐levels, which, in turn, will translate into sensitivity toward this parameter (Fig. 6).

We investigated the reproducibility of several subtypes of GLCM entropy feature as they were reported as one the highest reproducible and predictive radiomic features.^21^, ^27^, ^30^ We included: *Entropy, Summation Entropy, Difference Entropy* in addition to *first‐order Entropy (Intensity Histogram Entropy)*. GLCM entropy‐based features were strongly affected by the high heterogeneity of cervical cancer tumors as they measure the degree of nonuniformity within a given region of interest.

SRE and RPC, GLRLM regional features, showed the highest reproducibility through all testing parameters. This result can be explained by the fact that SRE only measures the distribution of short runs in the image (region) texture without taking into account gray‐level intensity.^46^ The high reproducibility of RPC (measures the homogeneity and the distribution of runs of an image in a specific direction) can be explained by the fact that gray‐level discretization does not highly impact the homogeneity of the run.

On the contrary, most of the regional features calculated based on GLSZM showed sensitivity to all testing parameters. These features may be categorized into different subsets. Features that focus on small homogenous and low‐intensity areas within the tumor volume, SAE, LIE, and LISAE, showed high sensitivity to variation in gray‐levels. This subset was the lowest reproducible among all features within the scope of this study (L/URL: ±100%–200%).

On the other hand, GLSZM features subset that characterizes large homogeneous and high‐intensity areas had a slightly better reproducibility range (L/URL: ±55%–90%). However, it was still lower than our proposed acceptable reproducibility limits. As previously mentioned, cervical tumors are associated with high regional FDG uptake, which might be the reason they perform slightly better than the other subset. Also, Tixier et al^37^ reported that high‐intensity areas correspond to aggressive tumor regions associated with high ^18^F‐FDG uptake while the large homogeneous area is thought to be less likely affected by statistical noise or partial‐volume effects. NGDTM features performed similarly to GLSZM features even after correction for volume and gray‐level dependency.

On a similar note, all of the IVH global features tested in this study (e.g., mean, SD, and kurtosis) showed a sensitivity toward all testing parameters. This outcome was expected because, on the one hand, they have large variations due to their lack of measuring significant information of uptake heterogeneity within the given tumor volume, and on the other hand, because of the delicate method used to calculate such features. We extracted shape‐based features (SF) to illustrate the morphological characteristics describing the voxel intensity distribution within the segmented tumor volumes without taking into consideration spatial relationships between neighboring voxels. As we fixed the volume tested for discretization, all SF showed insensitivity toward gray‐level discretization. Finally, the method described by Shafiq et al did not reflect similar results on PET images for most of the corrected radiomic features. However, GLCM features showed higher reproducibility after correction for volume and gray‐level dependence (Table S2a).

Variations introduced by reconstruction algorithms are different for each scanner vendor. These differences add difficulties in comparing results across institutions with different scanners. Along the same vein, it creates challenges to generate large patient cohort with similar clinical setups. Fortunately, despite this variation, different vendors produced reconstruction algorithms that are similar enough to be quantitatively comparable. According to our results, most of the radiomic features rely heavily on the choice of image reconstruction algorithm, whereas 3DRP had the least reproducible outputs.

**Figure 9:**
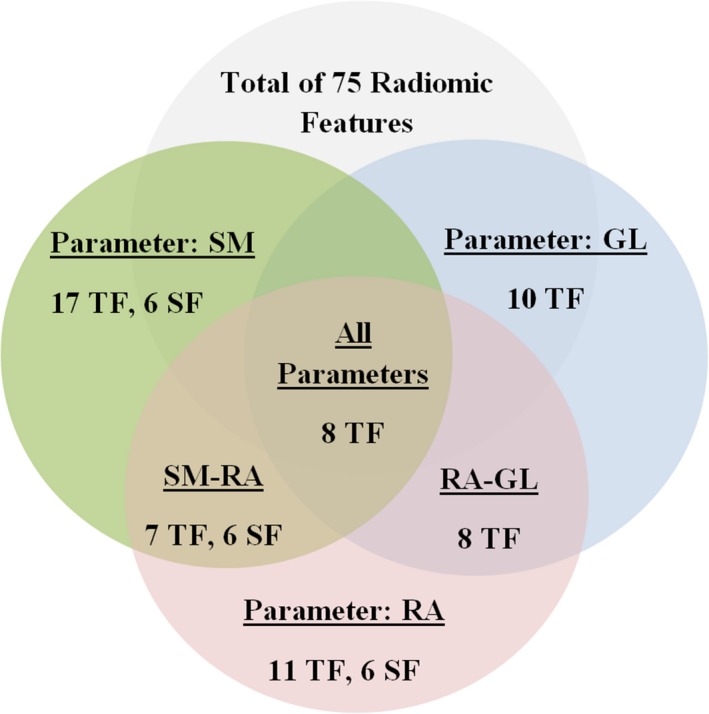
A diagram presenting the number of reproducible radiomic features per testing parameter and the common reproducible featured among all tests. The features are categorized into textural and shape features (TF and SF, respectively). The reproducibility through the three‐dimensional reprojection (3DRP) reconstruction algorithm is excluded. SM: Segmentation Method, GL: Gray Level, and RA: Reconstruction Algorithm.

Standardization and robustness are of utmost importance in this field; we suggest that features characterized by insensitivity toward segmentation methods, gray intensity level, and reconstruction algorithms (Fig. 9) may contribute as a robust characterizing descriptor of ^18^F‐FDG uptake heterogeneity and, therefore, might have promising clinical potential. However, such features might not demonstrate the same reproducibility in other tumor sites. This site‐specific study underlines the need for a profound analysis of radiomic features as descriptors of ^18^F‐FDG PET heterogeneity in cervical cancer patients treated with definitive radiochemotherapy. Accordingly, other site‐specific radiomic studies are required to examine the reproducibility of the mentioned features in a different tumor site

The relatively small cohort of patients might be a limitation of the current study. However, this cohort is about the same size, or larger, in comparison to samples in previously published reproducibility studies. Finally, although it is a challenging task, we support multicenter collaborative efforts that aim to standardize the process of radiomic analysis.

## 5 CONCLUSION

This study examined the reproducibility of several radiomic features extracted from ^18^F^−^FDG PET images of cervical cancer patients in response to the variation of three parameters: segmentation methods, gray intensity levels, and reconstruction algorithms. According to our results, most of the radiomic features within the scope of this study were highly affected by variations of such parameters. Therefore, we suggest that testing the reproducibility of radiomic features is essential before proceeding to employ them in any clinical applications.

## CONFLICT OF INTEREST

The authors declare no conflict of interest.

## Supporting information


**Table S1.** Heatmap illustrating the Dice coefficient (DC) for manual and semiautomatic methods. The volumes are sorted from small to large based on MTV1. A perfect overlap between pairs of tumor volumes is indicated by a DC value of 1.0. Acceptance Criteria: DC≥ 0.75 for all tumor pairs.

**Table S2.** Descriptive Statistics for Mean Percentage Difference (d) measured for Segmentation Method (SM) pairs of: 1) MTV1‐GBSV and 2) MTV2‐GBSV MTV1: First Manually Segmented Metabolic Tumor Volume, Reference Volume. MTV2: Second Manually Segmented Metabolic Tumor Volume, Reference Volume. GBSV: Graphical‐Based (Region‐Based) Semiautomatic volume.

**Table S3.** Descriptive Statistics for Mean Percentage Difference (d) measured for Gray Intensity Levels (GL) pairs of: 1) 64‐32 , 2) 64‐128, and 3) 64‐256.

**Table S4.** Descriptive Statistics for Mean Percentage Difference (d) measured for Gray Intensity Levels (GL) pairs of: 1) 64‐32 , 2) 64‐128, and 3) 64‐256.
