# Supplementary material for: Reproducibility of F18‐FDG PET radiomic features for different cervical tumor segmentation methods, gray‐level discretization, and reconstruction algorithms
Source: J Appl Clin Med Phys. 2017 Sep 11;18(6):32–48. doi: 10.1002/acm2.12170 (PMC5689938; doi:10.1002/acm2.12170)
Supplement: Supplementary file 1 — Table S1. Heatmap illustrating the Dice coefficient (DC) for manual and semiautomatic methods. The volumes are sorted from small to large based on MTV1. A perfect overlap between pairs of tumor volumes is indicated by a DC value of 1.0. Acceptance Criteria: DC≥ 0.75 for all tumor pairs. [file ACM2-18-032-s001.pdf]

Supplementary Document Page 1

Table1: Heatmap illustrating the dice coefficient (DC) for manual and semi-autmatic methods. The volumes are sorted from small to large based on MTV1. A perfect overlap between pairs of tumor volumes is indicated by a DC value of 1.0. Acceptance Criteria: DC≥ 0.75 for all tumor pairs.

Case No.	MTV1	MTV2	GBSV	MTV1-MTV2	MTV1-GBSV	MTV2-GBSV	DC > 0.75 (y/n)
1	8.90	8.70	27.48	0.91	0.48	0.45	n
2	9.29	8.90	14.38	0.93	0.61	0.58	n
3	9.98	11.64	16.63	0.73	0.65	0.69	n
4	12.17	5.87	7.92	0.44	0.50	0.69	n
5	12.62	31.78	14.17	0.53	0.69	0.54	n
6	13.10	15.94	17.02	0.71	0.80	0.88	y
7	14.47	8.12	9.88	0.68	0.60	0.74	n
8	16.86	14.69	16.43	0.94	0.84	0.85	y
9	17.99	24.74	22.88	0.75	0.79	0.91	y
10	19.27	24.64	23.47	0.75	0.88	0.85	y
11	20.53	20.24	22.08	0.97	0.95	0.92	y
12	20.83	20.24	22.20	0.98	0.91	0.90	y
13	21.71	22.49	17.51	0.92	0.72	0.76	y
14	21.71	22.69	29.63	0.91	0.74	0.76	y
15	22.00	19.95	17.33	0.91	0.78	0.88	y
16	22.30	26.60	30.51	0.86	0.83	0.88	y
17	24.84	25.72	22.69	0.96	0.75	0.84	y
18	26.40	35.30	29.44	0.77	0.90	0.90	y
19	30.12	33.64	31.89	0.85	0.89	0.80	y
20	30.90	29.53	26.00	0.95	0.77	0.80	y
21	31.49	55.16	79.70	0.62	0.70	0.69	n
22	32.86	37.77	43.64	0.92	0.82	0.75	y
23	33.25	35.00	41.76	0.92	0.76	0.80	y
24	36.28	39.80	42.25	0.88	0.88	0.92	y
25	38.04	39.41	33.15	0.94	0.88	0.85	y
26	39.80	45.57	42.35	0.83	0.92	0.93	y
27	41.86	55.16	55.35	0.87	0.83	0.92	y
28	42.64	39.31	48.31	0.78	0.89	0.84	y
29	43.00	47.04	55.65	0.88	0.86	0.86	y
30	43.68	52.71	53.40	0.75	0.85	0.90	y
31	44.73	51.29	51.85	0.85	0.91	0.93	y
32	44.81	52.16	65.81	0.75	0.77	0.87	y
33	45.28	53.89	63.76	0.79	0.83	0.88	y
34	49.39	54.28	52.00	0.88	0.82	0.83	y
35	54.24	66.89	71.06	0.75	0.86	0.92	y
36	55.00	59.66	56.82	0.90	0.77	0.74	y
37	55.16	66.50	56.00	0.78	0.91	0.83	y
38	57.55	69.47	71.59	0.78	0.79	0.80	y
39	60.24	66.00	70.00	0.89	0.87	0.82	y
40	64.89	79.02	71.76	0.79	0.93	0.89	y
41	66.01	54.28	56.72	0.79	0.83	0.85	y
42	67.58	71.00	56.72	0.93	0.73	0.82	y
43	69.00	66.70	68.75	0.93	0.92	0.87	y

DC range

0.00
0.25
0.50
0.75
1.00

44	69.63	171.22	118.63	0.56	0.55	0.69	n
45	72.57	89.78	71.49	0.76	0.88	0.86	y
46	74.33	94.12	90.69	0.76	0.88	0.91	y
47	76.38	79.83	74.23	0.94	0.93	0.92	y
48	78.15	82.54	98.80	0.87	0.81	0.90	y
49	79.22	160.88	133.69	0.43	0.63	0.69	n
50	83.10	81.06	95.40	0.96	0.88	0.90	y
51	84.79	90.66	82.00	0.93	0.84	0.90	y
52	87.63	106.01	95.84	0.79	0.82	0.81	y
53	89.39	88.00	119.80	0.98	0.85	0.83	y
54	92.12	102.00	93.00	0.88	0.87	0.87	y
55	92.51	105.33	88.00	0.85	0.89	0.90	y
56	98.18	116.63	109.00	0.76	0.92	0.83	y
57	98.68	115.30	106.60	0.82	0.87	0.87	y
58	101.06	82.84	121.92	0.80	0.78	0.76	y
59	104.93	118.05	120.19	0.87	0.89	0.89	y
60	107.00	112.00	120.88	0.94	0.82	0.93	y
61	108.36	98.32	98.36	0.89	0.93	0.94	y
62	109.92	136.52	131.48	0.75	0.89	0.90	y
63	119.02	130.00	125.00	0.90	0.79	0.74	y
64	124.10	149.53	130.00	0.80	0.85	0.80	y
65	124.64	191.19	107.48	0.46	0.68	0.55	n
66	128.61	151.57	149.63	0.82	0.80	0.79	y
67	129.09	289.67	197.75	0.40	0.73	0.73	n
68	130.36	147.18	145.52	0.86	0.83	0.86	y
69	140.69	140.52	174.12	0.99	0.90	0.89	y
70	143.27	169.57	160.68	0.82	0.84	0.83	y
71	146.70	157.81	134.47	0.92	0.89	0.82	y
72	151.29	188.17	161.07	0.75	0.93	0.83	y
73	157.16	274.12	237.84	0.56	0.69	0.68	n
74	162.00	153.64	154.91	0.85	0.79	0.82	y
75	163.91	324.20	129.68	0.42	0.62	0.53	n
76	165.08	199.80	183.27	0.78	0.93	0.92	y
77	169.52	191.64	120.00	0.86	0.74	0.70	n
78	178.87	187.48	166.04	0.95	0.80	0.77	y
79	184.45	164.59	158.31	0.82	0.75	0.76	y
80	248.40	383.26	131.05	0.45	0.59	0.50	n
