# Supplementary material for: Reproducibility of F18‐FDG PET radiomic features for different cervical tumor segmentation methods, gray‐level discretization, and reconstruction algorithms
Source: J Appl Clin Med Phys. 2017 Sep 11;18(6):32–48. doi: 10.1002/acm2.12170 (PMC5689938; doi:10.1002/acm2.12170)
Supplement: Supplementary file 2 — Table S2. Descriptive Statistics for Mean Percentage Difference (d) measured for Segmentation Method (SM) pairs of: 1) MTV1‐GBSV and 2) MTV2‐GBSV MTV1: First Manually Segmented Metabolic Tumor Volume, Reference Volume. MTV2: Second Manually Segmented Metabolic Tumor Volume, Reference Volume. GBSV: Graphical‐Based (Region‐Based) Semiautomatic volume. [file ACM2-18-032-s002.pdf]

Supplementary Document Page 2

Table 2: Descriptive Statistics for Mean Percentage Difference (d) measured for Segmentation Method (SM) pairs of: 1) MTV1-GBSV, and 2) MTV2-GBSV
MTV1: First Manually Segmented Metabolic Tumor Volume, Reference Volume.
MTV2: Second Manually Segmented Metabolic Tumor Volume, Reference Volume.
GBSV: Graphical-Based (Region-Based) Semi-automatic volume

Radiomic features calc. method	d%	SD	Reproducibility Level (RL)
**A) GLCM Features**	Statistic	Statistic	High/Med/Low/NR
_autocorrelation_MTV1_GBSV	7.61	33.57	NR
_autocorrelation_MTV2_GBSV	12.11	35.34	
_Cluster_prominence_MTV1_GBSV	-17.60	37.98	
_Cluster_prominence_MTV2_GBSV	-3.98	38.81	
_Cluster_Shade_MTV1_GBSV	6.18	36.66	
_Cluster_Shade_MTV2_GBSV	2.73	39.87	
_Cluster_tendency_MTV1_GBSV	-12.43	31.60	NR
_Cluster_tendency_MTV2_GBSV	-2.35	31.07	
_Contrast_MTV1_GBSV	0.07	35.01	NR
_Contrast_MTV2_GBSV	19.45	31.37	
_Correlation_MTV1_GBSV	-11.77	40.86	
_Correlation_MTV2_GBSV	-16.01	41.69	
**_Difference_Average_MTV1_GBSV**	2.60	19.11	High
**_Difference_Average_MTV2_GBSV**	4.58	22.53	
**_Difference_Entropy_MTV1_GBSV**	-2.16	17.76	High
**_Difference_Entropy_MTV2_GBSV**	-2.38	13.68	
**_Difference_Variance_MTV1_GBSV**	1.52	18.94	Low
**_Difference_Variance_MTV2_GBSV**	1.60	25.08	
**_dissimilarity_MTV1_GBSV**	12.05	23.72	Low
**_dissimilarity_MTV2_GBSV**	10.94	31.69	
_energy_MTV1_GBSV	-0.66	12.57	NR
_energy_MTV2_GBSV	-10.37	10.50	
**_Entropy_MTV1_GBSV**	-0.23	29.05	High
**_Entropy_MTV2_GBSV**	1.03	26.60	
**_Gnorm_Entropy_MTV1_GBSV**	-0.03	18.43	High
**_Gnorm_Entropy_MTV2_GBSV**	0.79	15.01	
**_Homogeneity1_MTV1_GBSV**	-0.80	23.49	Med
**_Homogeneity1_MTV2_GBSV**	-8.37	20.05	
_Info_Correlation1_MTV1_GBSV	-8.37	8.40	NR
_Info_Correlation1_MTV2_GBSV	-3.02	8.49	
**_Info_Correlation2_MTV1_GBSV**	-3.37	6.61	High
**_Info_Correlation2_MTV2_GBSV**	-3.02	10.21	
**_Inverse_difference_moment_MTV1_GBSV**	-0.03	2.11	High
**_Inverse_difference_moment_MTV2_GBSV**	-1.20	2.18	
**_Inverse_difference_MTV1_GBSV**	-0.10	1.61	High
**_Inverse_difference_MTV2_GBSV**	-2.20	5.41	
_Inverse_variance_MTV1_GBSV	-9.31	25.48	NR

Tables information

Sample size (n) = 65 patients
Reproduciblity criteria:
1) Check if: d<=30% and SD(d) <=35
2) Reproducibility Level (RL) as per the table below

Color code
Failed to meet criteria 1
Features meet criteria 1 and 2 with RL: High/Med/Low

Reproducibility Level (RL)	
High	RL <= 30
Med	30< RL < =45
Low	45 < RL <= 50
NR*	RL > 50

NR: Not reproducible

	High	Med	Low	NR
MTV1-GBSV	17	4	3	55
MTV2-GBSV				

_Inverse_variance_MTV2_GBSV	-33.89	24.57	NR
_Inverse_Variance_P_MTV1_GBSV	-1.76	25.35	NR
_Inverse_Variance_P_MTV2_GBSV	-12.97	21.68	
_Vnorm_Inverse_Variance_P_MTV1_GBSV	-3.68	42.06	NR
_Vnorm_Inverse_Variance_P_MTV2_GBSV	-16.64	32.97	
_Local_homogeneity_MTV1_GBSV	-2.03	44.80	NR
_Local_homogeneity_MTV2_GBSV	-12.64	47.61	
_Max_Probability_MTV1_GBSV	-6.30	21.32	NR
_Max_Probability_MTV2_GBSV	-11.47	19.87	
_Mean_MTV1_GBSV	-7.07	12.28	NR
_Mean_MTV2_GBSV	-25.43	9.97	
**_Vnorm_Mean_MTV1_GBSV**	-1.11	31.14	Med
**_Vnorm_Mean_MTV2_GBSV**	-5.66	19.87	
**_VGnorm_Mean_MTV1_GBSV**	-2.08	3.57	High
**_VGnorm_Mean_MTV2_GBSV**	-3.85	2.54	
_Sum_Average_MTV1_GBSV	4.61	33.43	NR
_Sum_Average_MTV2_GBSV	8.39	30.11	
_Gnorm_Sum_Average_MTV1_GBSV	5.45	58.89	NR
_Gnorm_Sum_Average_MTV2_GBSV	7.43	59.42	
**_Sum_Entropy_MTV1_GBSV**	-0.54	25.15	High
**_Sum_Entropy_MTV2_GBSV**	1.08	14.52	
**_Gnorm_Sum_Entropy_MTV1_GBSV**	-0.34	11.20	High
**_Gnorm_Sum_Entropy_MTV2_GBSV**	0.79	15.31	
_Sum_Variance_MTV1_GBSV	-13.28	33.25	NR
_Sum_Variance_MTV2_GBSV	-2.25	29.89	
_Gnorm_Sum_Variance_MTV1_GBSV	-13.28	59.01	NR
_Gnorm_Sum_Variance_MTV2_GBSV	-2.25	52.30	
_Variance_MTV1_GBSV	0.82	42.58	
_Variance_MTV2_GBSV	-0.99	52.66	
_Vnorm_Variance_MTV1_GBSV	6.62	49.48	
_Vnorm_Variance_MTV2_GBSV	19.92	48.93	

Radiomic features calc. method	d%	SD	Reproducibility Level (RL)
**B) GLRLM features**	Statistic	Statistic	High/Med/Low/NR
**_SRE_MTV1_GBSV**	0.17	2.89	High
**_SRE_MTV2_GBSV**	0.42	2.24	
_SRHGE_MTV1_GBSV	8.31	7.64	NR
_SRHGE_MTV2_GBSV	12.30	8.51	
_SRLGE_MTV1_GBSV	7.41	7.10	NR
_SRLGE_MTV2_GBSV	24.90	7.59	
_RLNU_MTV1_GBSV	-7.58	8.45	NR
_RLNU_MTV2_GBSV	-19.36	7.48	
_Vnorm_RLNU_MTV1_GBSV	-0.78	7.70	NR
_Vnorm_RLNU_MTV2_GBSV	-0.84	8.10	
_GLNU_MTV1_GBSV	-7.76	10.69	NR
_GLNU_MTV2_GBSV	-24.47	10.18	

_Gnorm_GLNU_MTV1_GBSV	-8.82	11.18	NR
_Gnorm_GLNU_MTV2_GBSV	-14.26	11.89	
_Vnorm_GLNU_MTV1_GBSV	-1.29	11.58	NR
_Vnorm_GLNU_MTV2_GBSV	-16.55	10.71	
_VGnorm_GLNU_MTV1_GBSV	-2.14	6.65	NR
_VGnorm_GLNU_MTV2_GBSV	5.12	7.29	
_HGRE_MTV1_GBSV	7.38	5.01	NR
_HGRE_MTV2_GBSV	12.10	4.42	
_Gnorm_HGRE_MTV1_GBSV	8.29	2.52	NR
_Gnorm_HGRE_MTV2_GBSV	24.85	2.55	
_LGRE_MTV1_GBSV	-44.42	-39.15	
_LGRE_MTV2_GBSV	33.18	-34.51	
**_LRE_MTV1_GBSV**	-0.79	7.39	High
**_LRE_MTV2_GBSV**	0.17	7.55	
_LRHGE_MTV1_GBSV	7.25	35.47	NR
_LRHGE_MTV2_GBSV	6.81	22.00	
_LRLGE_MTV1_GBSV	-48.05	-9.69	
_LRLGE_MTV2_GBSV	58.16	-7.31	
**_RPC_MTV1_GBSV**	-1.16	10.43	High
**_RPC_MTV2_GBSV**	0.92	8.09	

**Radiomic features calc. method**	d%	SD	Reproducibility Level (RL)
**C) GLSZM features**	Statistic	Statistic	High/Med/Low/NR
_HIE_MTV1_GBSV	3.15	33.56	NR
_HIE_MTV2_GBSV	1.00	47.59	
**_Gnorm_HIE_MTV1_GBSV**	4.30	19.57	Med
**_Gnorm_HIE_MTV2_GBSV**	-0.87	19.86	
_HILAE_MTV1_GBSV	-12.37	29.41	NR
_HILAE_MTV2_GBSV	-32.13	23.79	
_HISAE_MTV1_GBSV	11.46	32.01	NR
_HISAE_MTV2_GBSV	15.64	23.64	
_IV_MTV1_GBSV	9.33	32.27	NR
_IV_MTV2_GBSV	-22.94	23.91	
_LAE_MTV1_GBSV	-11.91	28.84	NR
_LAE_MTV2_GBSV	-35.06	29.62	
_LIE_MTV1_GBSV	12.25	27.56	NR
_LIE_MTV2_GBSV	-1.08	20.47	
_LILAE_MTV1_GBSV	-2.49	23.10	NR
_LILAE_MTV2_GBSV	-30.23	9.83	
_LISAE_MTV1_GBSV	9.76	22.27	NR
_LISAE_MTV2_GBSV	5.27	5.84	
_SAE_MTV1_GBSV	7.96	36.52	NR
_SAE_MTV2_GBSV	10.26	32.49	
_SZV_MTV1_GBSV	13.12	35.45	NR
_SZV_MTV2_GBSV	-3.26	22.83	

**_ZP_MTV1_GBSV**	3.31	30.19	Med
**_ZP_MTV2_GBSV**	7.35	25.21	

**Radiomic features calc. method**	d%	SD	Reproducibility Level (RL)
**D) NTGTM features**	Statistic	Statistic	High/Med/Low/NR
_Busyness_MTV1_GBSV	1.73	1.55	NR
_Busyness_MTV2_GBSV	-7.53	2.28	
_Coarseness_MTV1_GBSV	3.42	0.08	NR
_Coarseness_MTV2_GBSV	20.79	0.13	
_Vnorm_Coarseness_MTV1_GBSV	3.42	2.68	NR
_Vnorm_Coarseness_MTV2_GBSV	0.51	0.08	
_Complexity_MTV1_GBSV	-24.51	1.30	NR
_Complexity_MTV2_GBSV	-26.19	3.68	
_Gnorm_Complexity_MTV1_GBSV	-24.51	0.11	NR
_Gnorm_Complexity_MTV2_GBSV	-26.19	0.24	
_Contrast_MTV1_GBSV	7.61	3.99	NR
_Contrast_MTV2_GBSV	19.72	0.74	
_Gnorm_Contrast_MTV1_GBSV	4.05	0.22	NR
_Gnorm_Contrast_MTV2_GBSV	20.28	1.32	
_Texture Strength_MTV1_GBSV	-30.94	0.08	NR
_Texture Strength_MTV2_GBSV	-11.06	0.28	
_Vnorm_Texture Strength_MTV1_GBSV	-34.18	3.01	NR
_Vnorm_Texture Strength_MTV2_GBSV	-29.37	0.24	
_VGnorm_Texture Strength_MTV1_GBSV	-28.16	0.79	NR
_VGnorm_Texture Strength_MTV2_GBSV	-33.24	3.99	

**Radiomic features calc. method**	d%	SD	Reproducibility Level (RL)
**E) Shape-Based Features**	Statistic	Statistic	High/Med/Low/NR
**_sph_disprop_MTV1_GBSV**	6.31	3.89	High
**_sph_disprop_MTV2_GBSV**	5.01	3.50	
**_sphericity_MTV1_GBSV**	-5.63	6.85	High
**_sphericity_MTV2_GBSV**	-4.97	6.91	
**_Surf_Acm2_MTV1_GBSV**	-1.15	5.25	High
**_Surf_Acm2_MTV2_GBSV**	-9.45	5.18	
_surf_ovr_vol_MTV1_GBSV	8.95	4.79	Low
_surf_ovr_vol_MTV2_GBSV	12.16	5.27	
**_convexity_MTV1_GBSV**	-5.53	3.87	High
**_convexity_MTV2_GBSV**	-4.93	3.47	
_compactness_MTV1_GBSV	-9.23	6.86	NR
_compactness_MTV2_GBSV	-15.15	6.96	

**Radiomic features calc. method**	d%	SD	Reproducibility Level (RL)
**F) IVH Features**	Statistic	Statistic	High/Med/Low/NR
_Intensity_contrast_MTV1_GBSV	-3.94	3.97	NR
_Intensity_contrast_MTV2_GBSV	-9.14	3.53	
_Intensity_energy_MTV1_GBSV	8.17	3.85	NR

_Intensity_energy_MTV2_GBSV	16.61	2.48	NR
**_Intensity_entropy_MTV1_GBSV**	-1.67	3.28	High
**_Intensity_entropy_MTV2_GBSV**	-3.04	2.74	
_Intensity_locall_homog_MTV1_GBSV	-2.48	5.53	NR
_Intensity_locall_homog_MTV2_GBSV	4.28	4.78	
_Intensity_uniformity_MTV1_GBSV	-1.42	5.34	NR
_Intensity_uniformity__MTV2_GBSV	-0.48	5.04	
_V10_V90_MTV1_GBSV	9.84	3.65	NR
_V10_V90_MTV2_GBSV	3.96	3.00	
_V40_MTV1_GBSV	11.84	2.13	NR
_V40_MTV2_GBSV	13.04	2.16	
_V70_MTV1_GBSV	11.32	3.20	NR
_V70_MTV2_GBSV	15.81	2.59	
_V80_MTV1_GBSV	10.64	3.48	NR
_V80_MTV2_GBSV	15.23	2.57	
_Maximum_Intensity_MTV1_GBSV	2.60	3.51	NR
_Maximum_Intensity_MTV2_GBSV	1.89	2.60	
_Mean_Intensity_MTV1_GBSV	8.50	3.13	NR
_Mean_Intensity_MTV2_GBSV	14.60	3.22	
_Peak_Intensity_MTV1_GBSV	5.57	3.00	NR
_Peak_Intensity_MTV2_GBSV	6.16	2.23	
_I10_I90_MTV1_GBSV	-0.03	3.50	NR
_I10_I90_MTV2_GBSV	-2.02	2.34	
_I30_MTV1_GBSV	6.70	3.64	NR
_I30_MTV2_GBSV	12.41	3.22	

**Radiomic features calc. method**	d%	SD	Reproducibility Level (RL)
**G) First order statistical Features**	Statistic	Statistic	High/Med/Low/NR
_SD_MTV1_GBSV	-0.60	10.46	NR
_SD_MTV2_GBSV	-2.70	11.12	
_Skewness_MTV1_GBSV	-76.66	10.83	NR
_Skewness_MTV2_GBSV	-31.95	10.01	
_Kurtosis_MTV1_GBSV	27.09	6.22	NR
_Kurtosis_MTV2_GBSV	-249.16	6.82	
_RMS_MTV1_GBSV	7.04	4.68	NR
_RMS_MTV2_GBSV	11.76	4.14	
_Coeff_Vari_MTV1_GBSV	-8.00	2.36	NR
_Coeff_Vari_MTV2_GBSV	-15.27	2.38	

V90 (volume percentage having at least intensity of 90%) and
I90 (minimum intensity of 90% of the highest intensity volume).
