# Supplementary material for: Reproducibility of F18‐FDG PET radiomic features for different cervical tumor segmentation methods, gray‐level discretization, and reconstruction algorithms
Source: J Appl Clin Med Phys. 2017 Sep 11;18(6):32–48. doi: 10.1002/acm2.12170 (PMC5689938; doi:10.1002/acm2.12170)
Supplement: Supplementary file 3 — Table S3. Descriptive Statistics for Mean Percentage Difference (d) measured for Gray Intensity Levels (GL) pairs of: 1) 64‐32 , 2) 64‐128, and 3) 64‐256. [file ACM2-18-032-s003.pdf]

Supplementary Document Page 3

Table 3) Descriptive Statistics for Mean Percentage Difference (d) measured for Gray Intensity Levels (GL) pairs of: 1) 64-32 , 2) 64-128, and 3) 64-256

**Radiomic features calc. method**	d%	SD	Reproducibility Level (RL)
**A) GLCM Features**	Statistic	Statistic	High/Med/Low/NR
_autocorrelation_32_64	-101.26	0.18	
_autocorrelation_64_128	-98.07	3.18	
_autocorrelation_64_256	-144.93	0.11	
_Cluster_prominence_32_64	-145.37	0.06	
_Cluster_prominence_64_128	-120.99	9.33	
_Cluster_prominence_64_256	-162.28	0.01	
_Cluster_Shade_32_64	-130.90	1.77	
_Cluster_Shade_64_128	-97.68	2.61	
_Cluster_Shade_64_256	-158.60	0.09	
_Cluster_tendency_32_64	-99.46	0.15	
_Cluster_tendency_64_128	-53.06	3.06	
_Cluster_tendency_64_256	-144.54	0.09	
_Contrast_32_64	42.56	1.49	
_Contrast_64_128	-95.51	4.21	
_Contrast_64_256	-144.02	0.12	
_Correlation_32_64	102.16	0.28	
_Correlation_64_128	69.27	4.57	
_Correlation_64_256	-142.07	0.84	
_Difference_Average_32_64	-55.01	0.31	
_Difference_Average_64_128	-54.19	1.65	
_Difference_Average_64_256	-98.00	0.16	
_Difference_Entropy_32_64	-14.70	0.67	High
_Difference_Entropy_64_128	10.61	0.98	High
_Difference_Entropy_64_256	-17.38	1.36	Med
_Difference_Variance_32_64	-98.52	0.25	
_Difference_Variance_64_128	-97.33	1.51	
_Difference_Variance_64_256	-144.14	0.09	
_dissimilarity_32_64	-11.22	0.32	
_dissimilarity_64_128	15.30	3.44	Low
_dissimilarity_64_256	-17.01	0.28	
_energy_32_64	29.82	0.90	
_energy_64_128	69.27	4.57	
_energy_64_256	-105.07	4.26	
_Entropy_32_64	-15.25	0.56	High
_Entropy_64_128	-10.90	0.93	High
_Entropy_64_256	-17.78	1.19	Med
_Homogeneity1_32_64	0.00	3.33	
_Homogeneity1_64_128	32.58	2.33	
_Homogeneity1_64_256	64.72	1.80	
_Info_Correlation1_32_64	-15.79	2.88	
_Info_Correlation1_64_128	-25.54	3.16	
_Info_Correlation1_64_256	52.25	4.63	
_Info_Correlation2_32_64	-11.41	1.10	
_Info_Correlation2_64_128	-11.64	0.97	Med

Tables information

Sample size (n) = 80 patients
Reproducibility is examined using:
1) Check if: d<=30% and SD(d) <=35
2) State Reproducibility Level (RL) as per the table below

Color code
Failed to meet criteria 1
Features meet criteria 1 and 2 with RL: High/Med/Low

Reproducibility Level (RL)	
High	RL <= 30
Med	30< RL < =45
Low	45 < RL <= 50
NR*	RL > 50

NR: Not reproducible

	High	Med	Low	NR
64-32	7	3	1	0
64-128	7	4	2	0
64-256	4	4	2	1

_Info_Correlation2_64_256	-19.74	1.52	
_Inverse_difference_32_64	-0.04	0.05	
_Inverse_difference_64_128	0.12	0.29	High
_Inverse_difference_64_256	0.08	0.02	
_Inverse_difference_moment_32_64	0.01	0.04	
_Inverse_difference_moment_64_128	0.20	0.14	High
_Inverse_difference_moment_64_256	0.15	0.01	
_Inverse_variance_32_64	38.28	3.17	
_Inverse_variance_64_128	40.59	5.01	
_Inverse_variance_64_256	80.30	4.40	
_Inverse_Variance_P_32_64	41.39	2.84	
_Inverse_Variance_P_64_128	43.27	3.98	
_Inverse_Variance_P_64_256	82.55	4.03	
_Local_homogeneity_64_32	0.00		
_Local_homogeneity_64_128	44.11	3.23	
_Local_homogeneity_64_256	83.13	2.67	
_max_Probability_32_64	67.63	3.04	
_max_Probability_64_128	55.55	4.75	
_max_Probability_64_256	94.39	4.19	
_Mean_32_64	35.95	7.45	
_Mean_64_128	-44.25	8.91	
_Mean_64_256	83.63	15.12	
_Vgnorm_Mean_32_64	0.93	15.35	Med
_Vgnorm_Mean_64_128	3.58	6.50	Med
_Vgnorm_Mean_64_256	6.08	10.73	Med
_Sum_Average_32_64	-55.74	0.49	
_Sum_Average_64_128	54.82	2.16	
_Sum_Average_64_256	98.37	0.60	
_Sum_Entropy_32_64	-15.07	0.49	High
_Sum_Entropy_64_128	-10.85	2.16	High
_Sum_Entropy_64_256	-17.64	0.60	Med
_Sum_Variance_32_64	-98.77	0.62	
_Sum_Variance_64_128	-97.82	0.95	
_Sum_Variance_64_256	-144.33	1.20	
_Variance_32_64	-69.06	5.60	
_Variance_64_128	110.61	5.68	
_Variance_64_256	149.35	6.61	

**Radiomic features calc. method**	d%	SD	Reproducibility Level (RL)
**B) GLRLM features**	Statistic	Statistic	High/Med/Low/NR
_LRE_32_64	7.89	1.63	
_LRE_64_128	12.88	2.10	Med
_LRE_64_256	12.85	2.12	
_LRHGE_32_64	-91.75	3.78	
_LRHGE_64_128	-91.92	4.57	
_LRHGE_64_256	-139.71	4.07	
_LRLGE_32_64	79.57	6.90	
_LRLGE_64_128	82.00	6.57	
_LRLGE_64_256	126.19	6.28	

_RLNU_32_64	-11.53	1.56	Med
_RLNU_64_128	-11.91	1.92	
_RLNU_64_256	7.58	2.97	
_RPC_32_64	-4.84	0.77	High
_RPC_64_128	-3.17	0.76	
_RPC_64_256	5.06	1.10	
_LGRE_32_64	72.09	6.48	
_LGRE_64_128	66.33	10.01	
_LGRE_64_256	121.00	6.29	
_GLNU_32_64	39.23	2.84	
_GLNU_64_128	38.01	4.36	
_GLNU_64_256	73.58	3.52	
_HGRE_32_64	-96.89	3.85	
_HGRE_64_128	94.27	4.76	
_HGRE_64_256	-141.06	4.10	
_SRE_32_64	-2.88	0.44	High
_SRE_64_128	-1.84	0.36	
_SRE_64_256	-2.99	0.48	
_SRHGE_32_64	-98.07	3.87	
_SRHGE_64_128	-94.84	4.80	
_SRHGE_64_256	-141.37	4.11	
_SRLGE_32_64	72.46	6.65	
_SRLGE_64_128	77.08	5.55	
_SRLGE_64_256	-119.06	6.36	

**Radiomic features calc. method**	d%	SD	Reproducibility Level (RL)
**C) GLSZM features**	Statistic	Statistic	High/Med/Low/NR
_HIE_32_64	-89.74	4.49	
_HIE_64_128	-89.70	3.96	
_HIE_64_256	-135.10	4.19	
_HILAE_32_64	-5.08	9.10	
_HILAE_64_128	-15.73	8.43	
_HILAE_64_256	-43.61	10.48	
_HISAE_32_64	-118.94	6.89	
_HISAE_64_128	114.04	6.29	
_HISAE_64_256	147.91	4.49	
_IV_32_64	5.24	6.47	
_IV_64_128	33.39	7.05	
_IV_64_256	63.59	7.40	
_LAE_32_64	82.58	7.79	
_LAE_64_128	74.45	8.26	
_LAE_64_256	107.55	7.06	
_LIE_32_64	40.25	10.29	
_LIE_64_128	48.00	10.27	
_LIE_64_256	89.39	9.99	
_LILAE_32_64	95.79	10.03	
_LILAE_64_128	85.68	10.62	
_LILAE_64_256	133.79	7.20	
_LISAE_32_64	-23.65	12.96	

_LISAE_64_128	-10.08	13.40	
_LISAE_64_256	36.42	13.99	
_SAE_32_64	-31.34	12.19	
_SAE_64_128	-46.76	8.04	
_SAE_64_256	-60.28	7.91	
_SZV_32_64	-48.53	0.64	
_SZV_64_128	-36.63	2.22	
_SZV_64_256	-56.21	0.25	
_ZP_32_64	-34.88	12.14	
_ZP_64_128	-19.91	2.25	
_ZP_64_256	-32.17	0.40	

**Radiomic features calc. method**	d%	SD	Reproducibility Level (RL)
**D) NTGTM features**	Statistic	Statistic	High/Med/Low/NR
_Busyness_32_64	138.17	222.81	
_Busyness_64_128	175.45	253.40	
_Busyness_64_256	220.36	1052.94	
_Coarseness_32_64	-45.38	-177.81	
_Coarseness_64_128	-34.25	-147.99	
_Coarseness_64_256	-52.56	-198.50	
_Vnorm_Coarseness_32_64	-39.68	-160.10	
_Vnorm_Coarseness_64_128	-29.94	-119.48	
_Vnorm_Coarseness_64_256	-45.96	-193.99	
_Complexity_32_64	-30.49	-121.65	
_Complexity_64_128	-17.41	-64.90	
_Complexity_64_256	-28.12	-176.79	
_Gnorm_Complexity_32_64	-91.70	52.06	
_Gnorm_Complexity_64_128	-88.68	-116.82	
_Gnorm_Complexity_64_256	-132.19	-176.16	
_Contrast_32_64	67.75	124.95	
_Contrast_64_128	72.07	84.73	
_Contrast_64_256	-111.33	-173.77	
_Gnorm_Contrast_32_64	-52.12	-67.28	
_Gnorm_Contrast_64_128	51.26	-66.28	
_Gnorm_Contrast_64_256	91.98	-119.86	
_Texture Strength_32_64	74.41	-17.98	
_Texture Strength_64_128	76.67	12.98	
_Texture Strength_64_256	118.00	-21.25	
_Vnorm_Texture Strength_32_64	63.24	-120.50	
_Vnorm_Texture Strength_64_128	51.95	-119.05	
_Vnorm_Texture Strength_64_256	88.26	-176.31	
_VGnorm_Texture Strength_32_64	89.72	-65.09	
_VGnorm_Texture Strength_64_128	-134.88	120.24	
_VGnorm_Texture Strength_64_256	134.30	-118.66	
