# Supplementary material for: Reproducibility of F18‐FDG PET radiomic features for different cervical tumor segmentation methods, gray‐level discretization, and reconstruction algorithms
Source: J Appl Clin Med Phys. 2017 Sep 11;18(6):32–48. doi: 10.1002/acm2.12170 (PMC5689938; doi:10.1002/acm2.12170)
Supplement: Supplementary file 4 — Table S4. Descriptive Statistics for Mean Percentage Difference (d) measured for Gray Intensity Levels (GL) pairs of: 1) 64‐32 , 2) 64‐128, and 3) 64‐256. [file ACM2-18-032-s004.pdf]

Table 4: Descriptive Statistics for Mean Percentage Difference (d) measured for Gray Intensity Levels (GL) pairs of: 1) 64-32 , 2) 64-128, and 3) 64-256

Radiomic features calc. method	(d) over n = 8	SD	Reproducibility Level (RL)
**A) GLCM features**	Statistic	**Statistic**	**High/Med/Low/NR**
_autocorrelation_OSEM_FOREIR	-25.92	3.70	
_autocorrelation_OSEM_FOREFBP	-31.79	4.06	NR
_autocorrelation_OSEM_3DRP	-17.71	5.96	
_Cluster_prominence_OSEM_FOREIR	-118.85	39.41	
_Cluster_prominence_OSEM_FOREFBP	-98.92	41.25	
_Cluster_prominence_OSEM_3DRP	-132.68	91.95	
_Cluster_Shade_OSEM_FOREIR	-107.02	40.07	
_Cluster_Shade_OSEM_FOREFBP	-79.86	67.37	
_Cluster_Shade_OSEM_3DRP	-129.67	80.02	
_Cluster_tendency_OSEM_FOREIR	-81.31	60.05	
_Cluster_tendency_OSEM_FOREFBP	-43.38	39.18	
_Cluster_tendency_OSEM_3DRP	-118.17	48.01	
**_Contrast_OSEM_FOREIR**	0.29	4.07	High
**_Contrast_OSEM_FOREFBP**	8.27	6.44	High
_Contrast_OSEM_3DRP	39.39	5.65	NR
_Correlation_OSEM_FOREIR	-5.94	3.65	
_Correlation_OSEM_FOREFBP	31.26	5.12	NR
_Correlation_OSEM_3DRP	38.09	5.18	
**_Difference_Average_OSEM_FOREIR**	-1.39	8.32	High
**_Difference_Average_OSEM_FOREFBP**	0.90	9.06	High
**_Difference_Average_OSEM_3DRP**	2.75	9.52	Med
**_Difference_Entropy_OSEM_FOREIR**	5.31	10.01	High
**_Difference_Entropy_OSEM_FOREFBP**	8.93	10.62	High
**_Difference_Entropy_OSEM_3DRP**	10.72	19.07	Med
**_Difference_Variance_OSEM_FOREIR**	0.57	8.34	High
**_Difference_Variance_OSEM_FOREFBP**	-0.21	12.87	High
**_Difference_Variance_OSEM_3DRP**	1.54	8.08	Med
**_dissimilarity_OSEM_FOREIR**	-0.95	13.23	High
**_dissimilarity_OSEM_FOREFBP**	-0.21	13.21	High
_dissimilarity_OSEM_3DRP	-16.01	12.85	NR
_energy_OSEM_FOREIR	19.15	12.90	
_energy_OSEM_FOREFBP	7.96	13.65	NR
_energy_OSEM_3DRP	-9.93	9.25	
**_Entropy_OSEM_FOREIR**	-1.44	16.66	High
**_Entropy_OSEM_FOREFBP**	-0.39	17.22	High
_Entropy_OSEM_3DRP	29.47	17.99	NR
**_Mean_OSEM_FOREIR**	-0.34	0.00	High
**_Mean_OSEM_FOREFBP**	-0.12	4.15	High
_Mean_OSEM_3DRP	29.00	3.44	NR
**_Vnorm_Mean_OSEM_FOREIR**	-0.29	2.10	High
**_Vnorm_Mean_OSEM_FOREFBP**	-4.91	2.70	High
**_Vnorm_Mean_OSEM_3DRP**	2.22	2.73	Med
**_Gnorm_Mean_OSEM_FOREIR**	-0.34	4.85	High
**_Gnorm_Mean_OSEM_FOREFBP**	-0.12	5.87	High

Tables information

Sample size (n) = 8 patients

Reproduciblity is examined using:

1) Check if: d<=30% and SD(d) <=35

2) State Reproducibility Level (RL) as per the table below

Color code

Failed to meet criteria 1 (excluded)

Features meet criteria 1 and 2 with RL: High/Med/Low

Reproducibility Level (RL)	
High	RL <= 30
Med	30< RL < =45
Low	45 < RL <= 50
NR*	RL > 50

NR: Not reproducible

	High	Med	Low	NR
OSEM-FORIR<br>OSEM-FOREFBP	21	1	4	13
OSEM-3DRP	0	9	4	26

_Gnorm_Mean_OSEM_3DRP	4.87	5.23	Med
_Vgnorm_Mean_OSEM_FOREIR	-0.29	8.87	High
_Vgnorm_Mean_OSEM_FOREFBP	-4.92	8.45	High
_Vgnorm_Mean_OSEM_3DRP	1.96	8.08	Med
_Homogeneity1_OSEM_FOREIR	4.26	3.91	High
_Homogeneity1_OSEM_FOREFBP	0.64	6.11	High
_Homogeneity1_OSEM_3DRP	28.57	5.38	NR
_Info_Correlation1_OSEM_FOREIR	-2.27	0.57	High
_Info_Correlation1_OSEM_FOREFBP	-3.07	1.17	High
_Info_Correlation1_OSEM_3DRP	5.06	0.15	Med
_Info_Correlation2_OSEM_FOREIR	-1.41	2.01	High
_Info_Correlation2_OSEM_FOREFBP	-1.29	2.47	High
_Info_Correlation2_OSEM_3DRP	4.96	3.82	Med
_Inverse_difference_OSEM_FOREIR	0.15	0.99	High
_Inverse_difference_OSEM_FOREFBP	-0.28	0.97	High
_Inverse_difference_OSEM_3DRP	4.33	1.42	Med
_Inverse_difference_moment_OSEM_FOREIR	-0.04	15.67	High
_Inverse_difference_moment_OSEM_FOREFBP	-0.31	10.34	High
_Inverse_difference_moment_OSEM_3DRP	-1.02	10.17	Med
_Inverse_variance_OSEM_FOREIR	8.91	0.56	High
_Inverse_variance_OSEM_FOREFBP	2.44	0.46	High
_Inverse_variance_OSEM_3DRP	31.46	6.61	NR
_Inverse_Variance_P_OSEM_FOREIR	-30.49	4.98	High
_Inverse_Variance_P_OSEM_FOREFBP	-17.41	6.17	High
_Inverse_Variance_P_OSEM_3DRP	-28.12	5.29	NR
_Local_homogeneity_64_32	-91.70	8.55	High
_Local_homogeneity_OSEM_FOREFBP	-88.68	7.13	High
_Local_homogeneity_OSEM_3DRP	-132.19	8.18	NR
_max_Probability_OSEM_FOREIR	77.88	0.63	
_max_Probability_OSEM_FOREFBP	82.45	2.77	NR
_max_Probability_OSEM_3DRP	-101.33	0.77	
_Mean_OSEM_FOREIR	-52.12	0.79	High
_Mean_OSEM_FOREFBP	51.26	1.22	High
_Mean_OSEM_3DRP	91.98	1.54	NR
_Sum_Average_OSEM_FOREIR	-48.53	12.41	
_Sum_Average_OSEM_FOREFBP	-36.63	20.26	
_Sum_Average_OSEM_3DRP	-56.21	20.20	
_Sum_Entropy_OSEM_FOREIR	-9.06	-14.29	High
_Sum_Entropy_OSEM_FOREFBP	10.61	-14.55	High
_Sum_Entropy_OSEM_3DRP	19.35	-29.70	Med
_Sum_Variance_OSEM_FOREIR	-34.88	125.14	
_Sum_Variance_OSEM_FOREFBP	-19.91	128.95	
_Sum_Variance_OSEM_3DRP	-32.17	198.45	
_Variance_OSEM_FOREIR	-2.88	106.36	
_Variance_OSEM_FOREFBP	-1.84	87.36	
_Variance_OSEM_3DRP	-2.99	148.44	

Radiomic features calc. method	d%	SD	Reproducibility Level (RL)
B) GLRLM features	Statistic	Statistic	High/Med/Low/NR

**_LRE_OSEM_FOREIR**	1.21	0.48	High
**_LRE_OSEM_FOREFBP**	0.83	3.37	High
_LRE_OSEM_3DRP	-23.04	8.18	NR
**_SRE_OSEM_FOREIR**	-0.09	0.98	High
**_SRE_OSEM_FOREFBP**	-0.20	6.69	High
_SRE_OSEM_3DRP	24.62	0.27	NR
_SRHGE_OSEM_FOREIR	-23.62	14.73	NR
_SRHGE_OSEM_FOREFBP	-27.95	5.27	NR
_SRHGE_OSEM_3DRP	-38.06	6.20	
_SRLGE_OSEM_FOREIR	-48.53	25.64	
_SRLGE_OSEM_FOREFBP	-36.63	12.47	
_SRLGE_OSEM_3DRP	-56.21	17.43	
_LRHGE_OSEM_FOREIR	-69.06	36.84	
_LRHGE_OSEM_FOREFBP	110.61	4.10	
_LRHGE_OSEM_3DRP	149.35	-1.50	
_LRLGE_OSEM_FOREIR	-34.88	37.85	
_LRLGE_OSEM_FOREFBP	-19.91	4.41	
_LRLGE_OSEM_3DRP	-32.17	-1.03	
_LGRE_OSEM_FOREIR	-98.77	38.65	
_LGRE_OSEM_FOREFBP	-97.82	5.68	
_LGRE_OSEM_3DRP	-144.33	3.54	
**_RLNU_OSEM_FOREIR**	-2.58	24.77	
**_RLNU_OSEM_FOREFBP**	-0.40	25.35	High
**_RLNU_OSEM_3DRP**	1.37	57.21	
_GLNU_OSEM_FOREIR	-13.57	-3.06	
_GLNU_OSEM_FOREFBP	6.59	-5.26	NR
_GLNU_OSEM_3DRP	14.43	-8.37	
_Gnorm_GLNU_OSEM_FOREIR	35.94	-19.89	
_Gnorm_GLNU_OSEM_FOREFBP	6.59	-24.71	NR
_Gnorm_GLNU_OSEM_3DRP	-2.23	-25.20	
_HGRE_OSEM_FOREIR	1.48	-27.22	NR
_HGRE_OSEM_FOREFBP	-28.37	-29.78	NR
_HGRE_OSEM_3DRP	-38.27	-31.04	
_Gnorm_HGRE_OSEM_FOREIR	0.93	-33.27	
_Gnorm_HGRE_OSEM_FOREFBP	-28.37	-37.20	NR
_Gnorm_HGRE_OSEM_3DRP	-9.22	-37.38	
**_RPC_OSEM_FOREIR**	-0.30	-8.24	High
**_RPC_OSEM_FOREFBP**	-0.22	-20.25	
_RPC_OSEM_3DRP	-25.61	-31.16	Med

**Radiomic features calc. method**	d%	**SD**	**Reproducibility Level (RL)**
**C) GLSZM features**	Statistic	**Statistic**	**High/Med/Low/NR**
_SAE_OSEM_FOREIR	-12.37	-0.44	
_SAE_OSEM_FOREFBP	-32.13	-3.36	NR
_SAE_OSEM_3DRP	11.46	-0.18	
_IV_OSEM_FOREIR	15.64	-0.57	
_IV_OSEM_FOREFBP	9.33	-6.89	NR
_IV_OSEM_3DRP	-22.94	-0.63	

_LAE_OSEM_FOREIR	-11.91	-5.46	
_LAE_OSEM_FOREFBP	-35.06	-11.26	NR
_LAE_OSEM_3DRP	12.25	-15.97	
_HIE_OSEM_FOREIR	-1.08	-10.13	
_HIE_OSEM_FOREFBP	-2.49	-22.85	
_HIE_OSEM_3DRP	-30.23	-34.01	
_HILAE_OSEM_FOREIR	9.76	11.44	
_HILAE_OSEM_FOREFBP	5.27	-38.93	
_HILAE_OSEM_3DRP	7.96	-55.35	
_HISAE_OSEM_FOREIR	10.26	10.08	
_HISAE_OSEM_FOREFBP	13.12	-39.14	
_HISAE_OSEM_3DRP	-3.26	-55.49	
_LIE_OSEM_FOREIR	3.42	7.71	
_LIE_OSEM_FOREFBP	0.51	-39.50	NR
_LIE_OSEM_3DRP	-24.51	-58.94	
_LILAE_OSEM_FOREIR	-26.19	-17.79	
_LILAE_OSEM_FOREFBP	-24.51	-64.16	
_LILAE_OSEM_3DRP	-26.19	-81.90	
_LISAE_OSEM_FOREIR	7.61	-6.99	
_LISAE_OSEM_FOREFBP	19.72	-41.12	
_LISAE_OSEM_3DRP	4.05	-66.69	
_SZV_OSEM_FOREIR	20.28	-69.67	
_SZV_OSEM_FOREFBP	-3.94	-25.10	NR
_SZV_OSEM_3DRP	-9.14	-28.30	
_ZP_OSEM_FOREIR	8.17	-55.49	
_ZP_OSEM_FOREFBP	16.61	-28.30	NR
_ZP_OSEM_3DRP	29.49	-28.30	

**Radiomic features calc. method**	d%	**SD**	**Reproducibility Level (RL)**
**D) NTGTM features**	Statistic	**Statistic**	**High/Med/Low/NR**
_Busyness_OSEM_FOREIR	-828.74	-532.70	
_Busyness_OSEM_FOREFBP	1427.29	-198.42	
_Busyness_3DRP	1601.65	-741.28	
_Coarseness_OSEM_FOREIR	5.82	-11.18	
_Coarseness_OSEM_FOREFBP	18.62	-30.09	Low
_Coarseness_3DRP	24.84	-2.68	
_Vnorm_Coarseness_OSEM_FOREIR	10.62	-4.95	
_Vnorm_Coarseness_OSEM_FOREFBP	18.58	5.92	Low
_Vnorm_Coarseness_3DRP	29.48	10.78	
_Complexity_OSEM_FOREIR	2.71	7.00	Low
_Complexity_OSEM_FOREFBP	1.59	9.55	Low
_Complexity_3DRP	-6.97	-0.70	NR
_Gnorm_Complexity_OSEM_FOREIR	-2.66	1.38	
_Gnorm_Complexity_OSEM_FOREFBP	-4.60	-0.44	NR
_Gnorm_Complexity_3DRP	17.62	1.02	
_Contrast_OSEM_FOREIR	-4.95	-17.08	Low
_Contrast_OSEM_FOREFBP	6.53	-2.89	Low
_Contrast_3DRP	-19.61	-17.08	NR

_Gnorm_Contrast_OSEM_FOREIR	-6.73	-2.89	Med
_Gnorm_Contrast_OSEM_FOREFBP	4.47	1.05	Med
_Gnorm_Contrast_3DRP	-19.01	-1.28	NR
_Texture Strength_OSEM_FOREIR	9.93	8.51	
_Texture Strength_OSEM_FOREFBP	31.55	25.62	NR
_Texture Strength_3DRP	38.91	0.22	
_VGnorm_Texture Strength_OSEM_FOREIR	15.27	-2.89	
_VGnorm_Texture Strength_OSEM_FOREFBP	19.66	-17.08	NR
_VGnorm_Texture Strength_3DRP	23.81	-2.89	
